# Supplementary material for: Network Pharmacology Revealing the Therapeutic Potential of Bioactive Components of Triphala and Their Molecular Mechanisms against Obesity
Source: Int J Mol Sci. 2024 Oct 6;25(19):10755. doi: 10.3390/ijms251910755 (PMC11476943; doi:10.3390/ijms251910755)
Supplement: Supplementary file 1 [file ijms-25-10755-s001.zip › ijms-3171983-supplementary.pdf]

Supplementary Material

Network Pharmacology Revealing the Therapeutic Potential of Bioactive Components of Triphala and Their Molecular Mechanisms against Obesity

Ratchanon Inpan [1,2,3], Chotiwit Sakuludomkan [1,2], Mingkwan Na Takuathung [1,2] and Nut Koonrungsesomboon [1,2,*]

1 Department of Pharmacology, Faculty of Medicine, Chiang Mai University, Chiang Mai 50200, Thailand; ratchanon.inpan@gmail.com or ratchanon.inpan@cmu.ac.th (R.I.); chotiwit.cs@gmail.com (C.S.); mingkwan.n@cmu.ac.th (M.N.)
2 Clinical Research Center for Food and Herbal Product Trials and Development (CR-FAH), Faculty of Medicine, Chiang Mai University, Chiang Mai 50200, Thailand
3 Office of Research Administration, Chiang Mai University, Chiang Mai 50200, Thailand
* Correspondence: nkoonrung@gmail.com or nut.koonrung@cmu.ac.th; Tel.: +66-53-935352

Supplementary Figure Legends

Supplementary Figure S1. Overview study diagram.

Supplementary Figure S2. The bond interaction residue and bond distance between beta-sitosterol compound and AKT1 protein.

Supplementary Figure S3. The bond interaction residue and bond distance between 7-dehydrosigmasterol compound and AKT1 protein.

Supplementary Figure S4. The bond interaction residue and bond distance between α-amyrin compound and AKT1 protein.

Supplementary Figure S5. The bond interaction residue and bond distance between peraksine compound and AKT1 protein.

Supplementary Figure S6. The bond interaction residue and bond distance between luteolin compound and AKT1 protein.

Supplementary Figure S7. The bond interaction residue and bond distance between quercetin compound and AKT1 protein.

Supplementary Figure S8. The bond interaction residue and bond distance between kaempferol compound and AKT1 protein.

Supplementary Figure S9. The bond interaction residue and bond distance between ellagic acid compound and AKT1 protein.

Supplementary Figure S10. The bond interaction residue and bond distance between 7-dehydrosigmasterol compound and PPARG protein.

Supplementary Figure S11. The bond interaction residue and bond distance between peraksine compound and PPARG protein.

Supplementary Figure S12. The bond interaction residue and bond distance between quercetin compound and PPARG protein.

Supplementary Figure S13. The bond interaction residue and bond distance between luteolin compound and PPARG protein.
Supplementary Figure S14. The bond interaction residue and bond distance between ellagic acid compound and PPARG protein.
Supplementary Figure S15. The bond interaction residue and bond distance between kaempferol compound and PPARG protein.
Supplementary Figure S16. The bond interaction residue and bond distance between phyllanthin compound and PPARG protein.

Supplementary Table Legends
Supplementary Table S1. Data of reported genes in obesity.
Supplementary Table S2. Data of overall Triphala target network construction.
Supplementary Table S3. Protein-protein interaction data of criteria of degree, betweenness, and closeness.
Supplementary Table S4. Data of Triphala compounds and genes target network construction.
Supplementary Table S5. Data of genes target and KEGG pathway network construction.

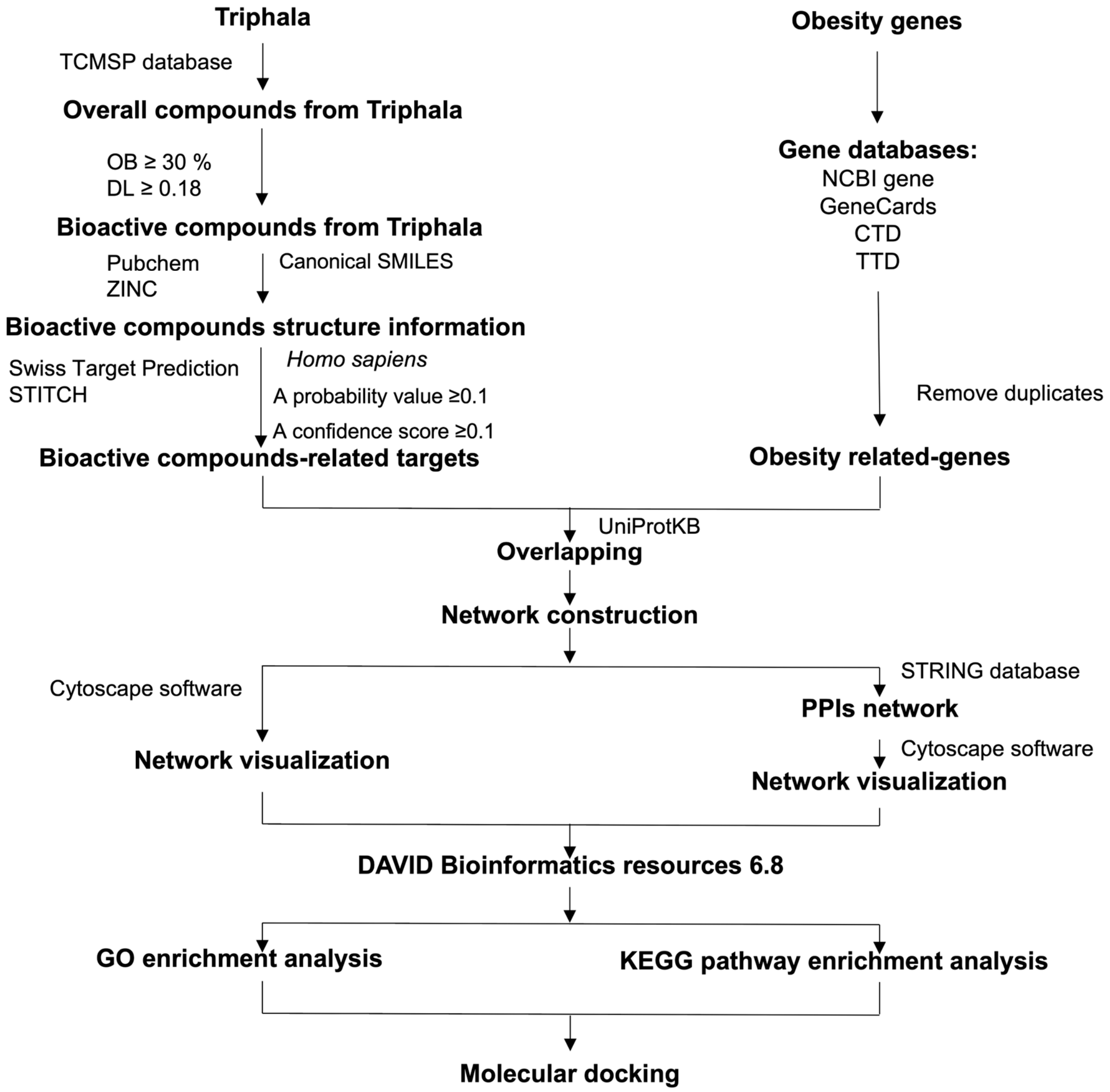

Supplementary Figure S1.

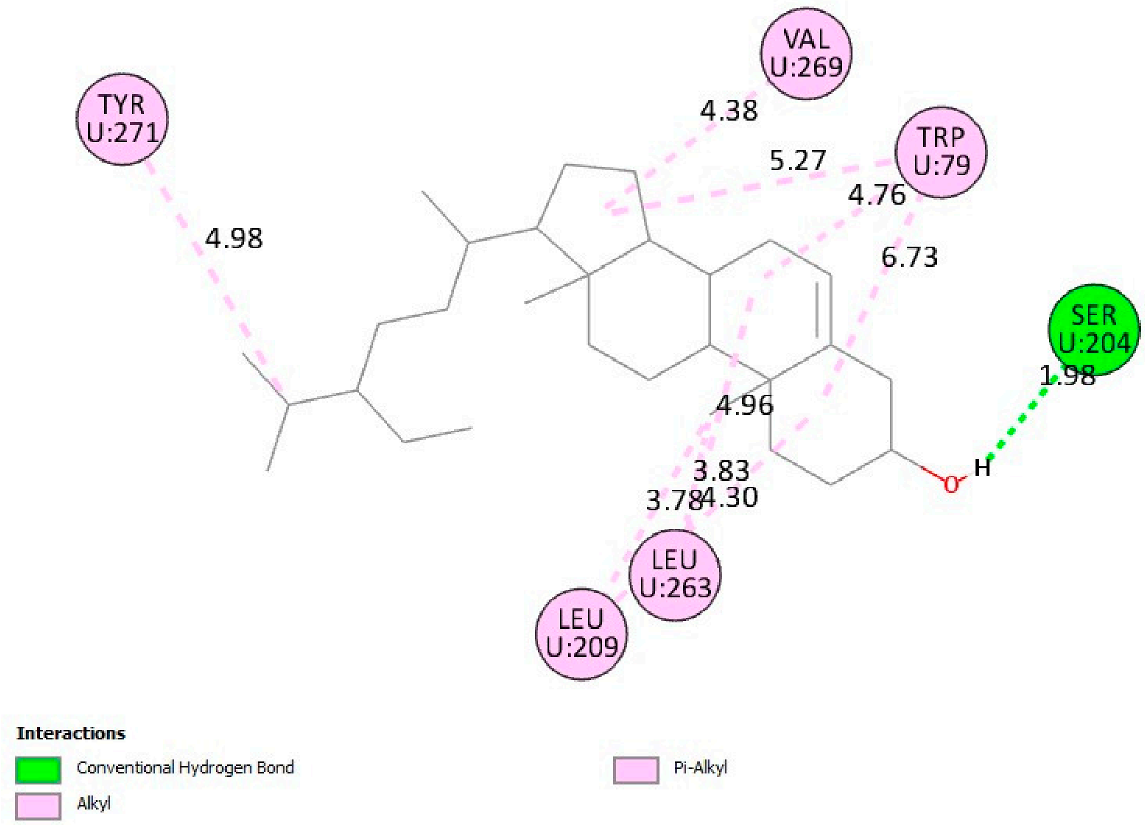

Supplementary Figure S2.

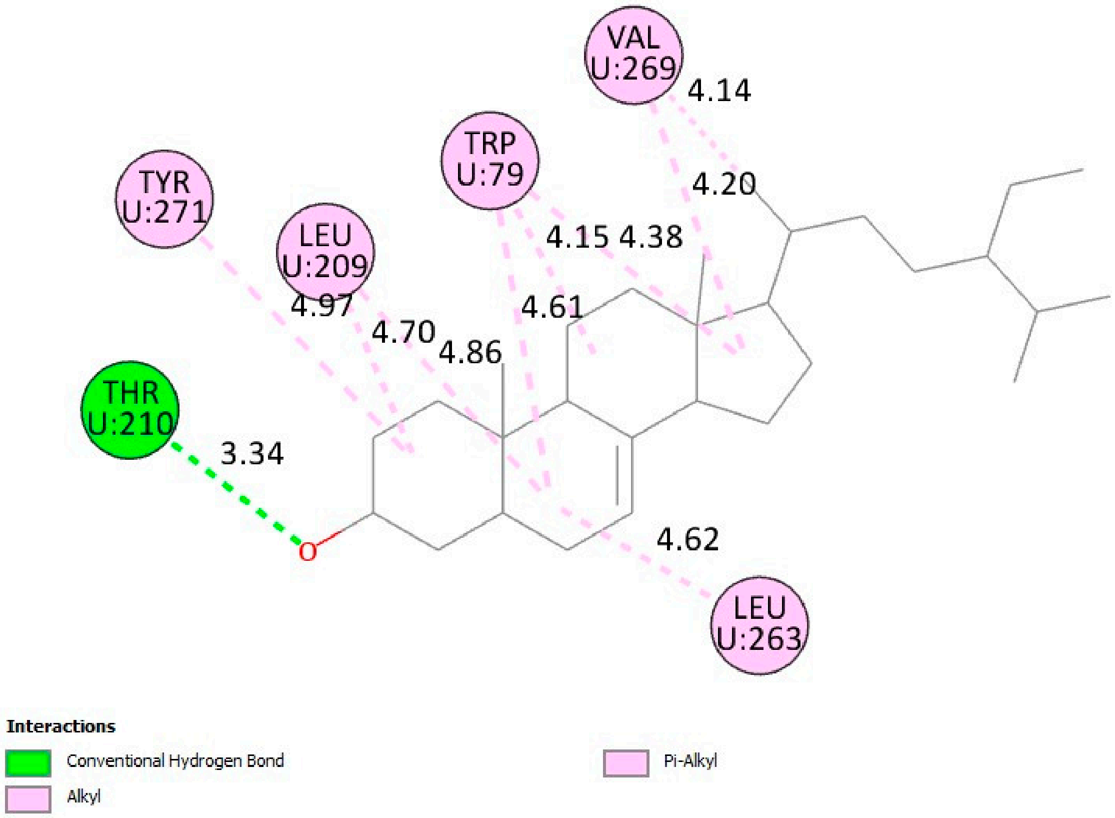

Supplementary Figure S3.

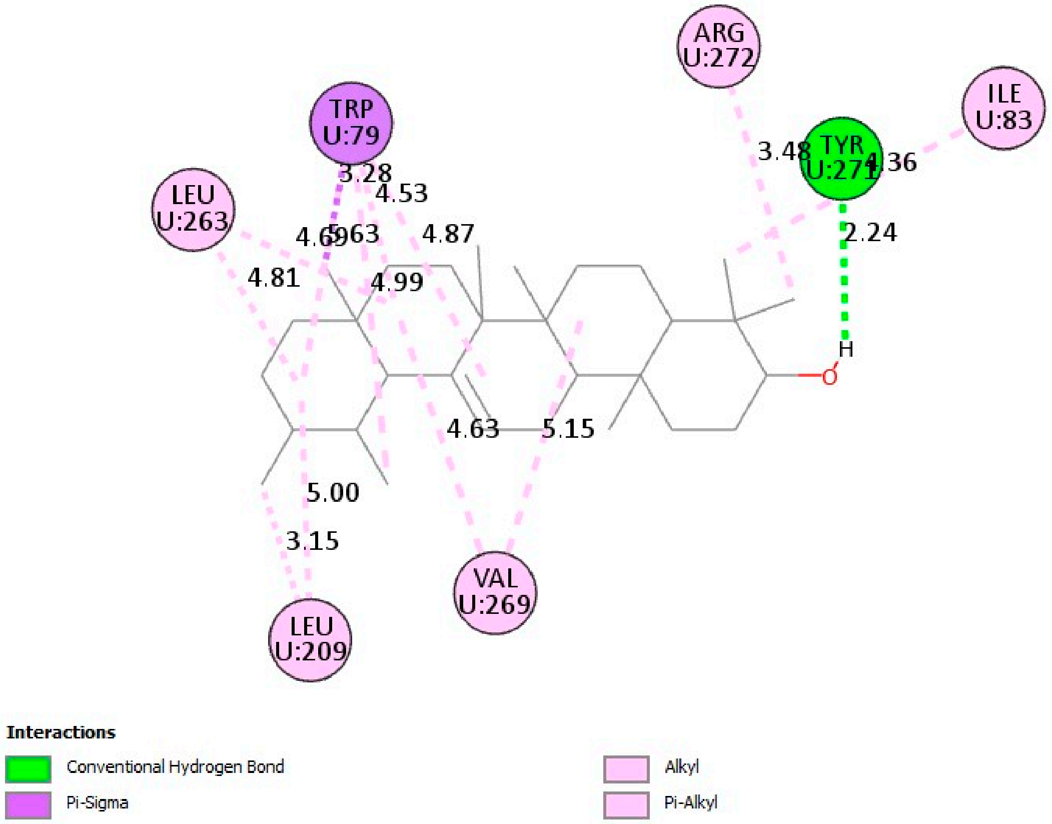

Supplementary Figure S4.

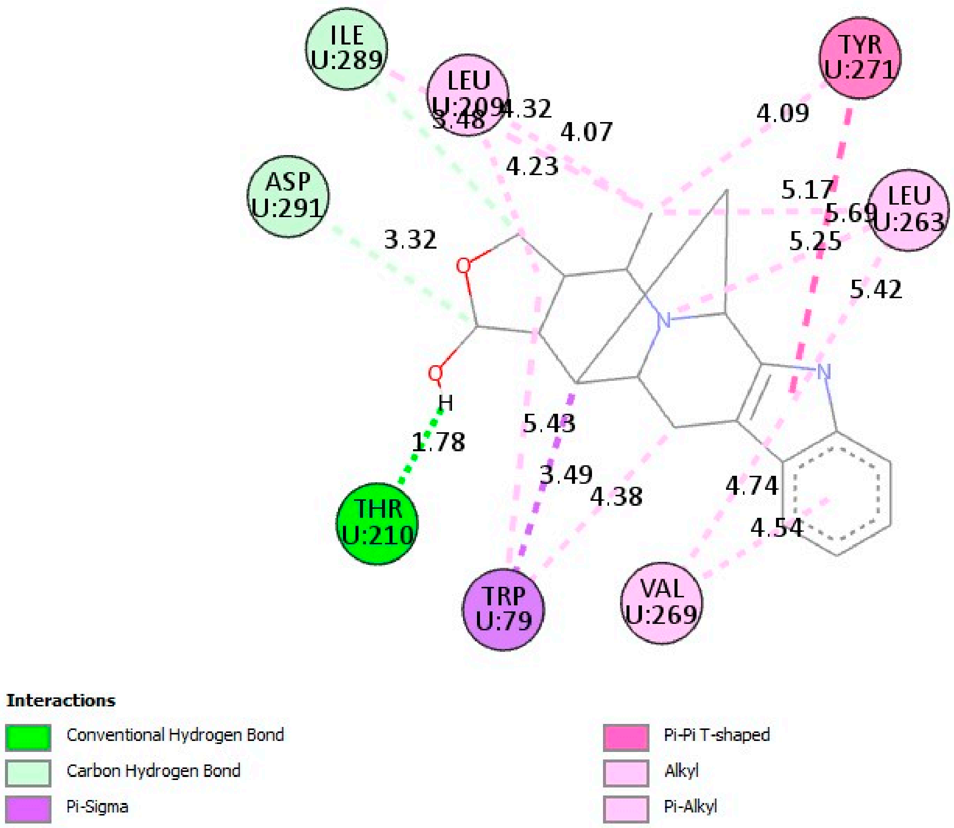

Supplementary Figure S5.

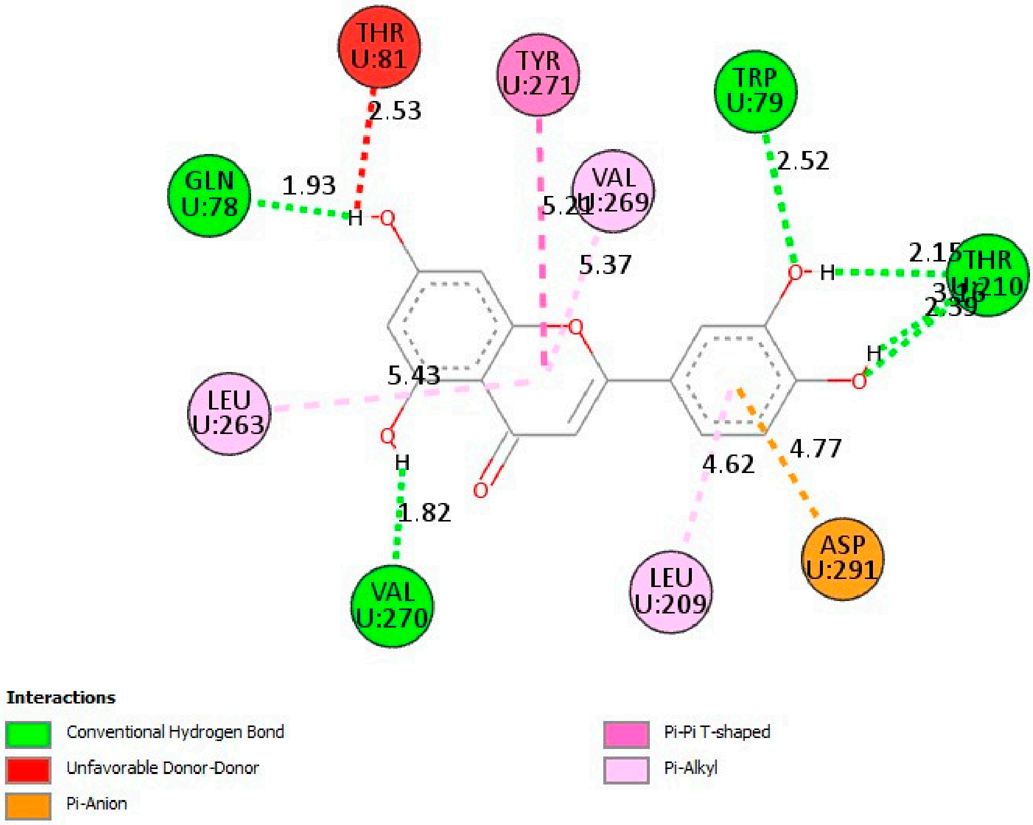

Supplementary Figure S6.

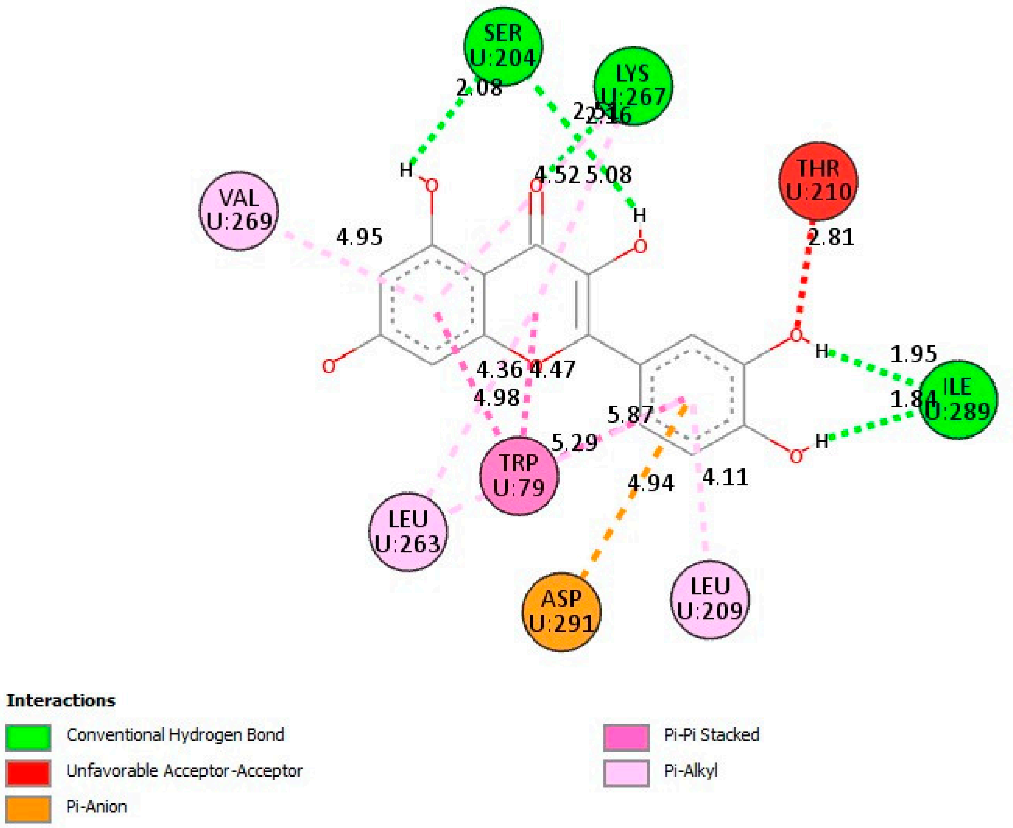

Supplementary Figure S7.

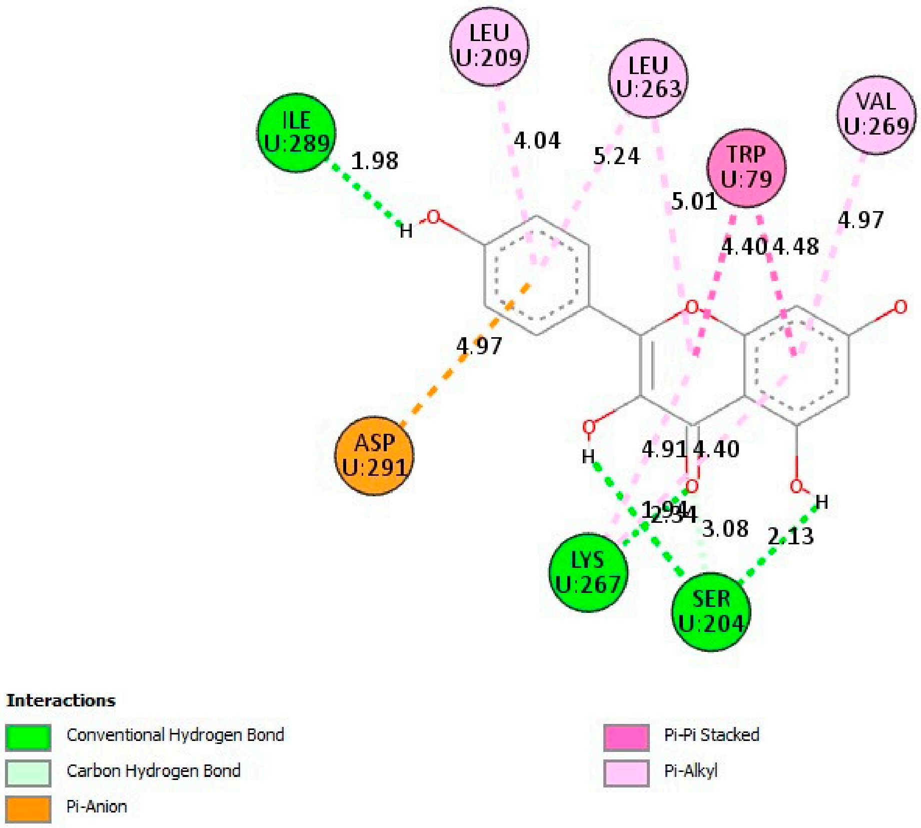

Supplementary Figure S8.

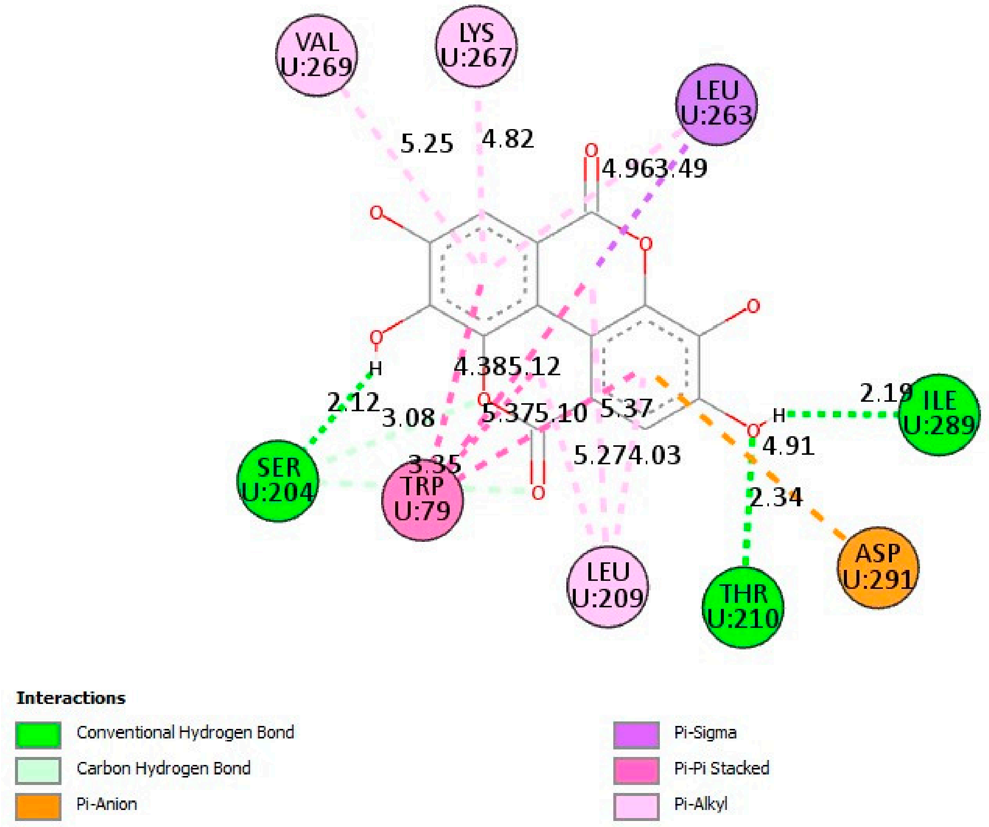

Supplementary Figure S9.

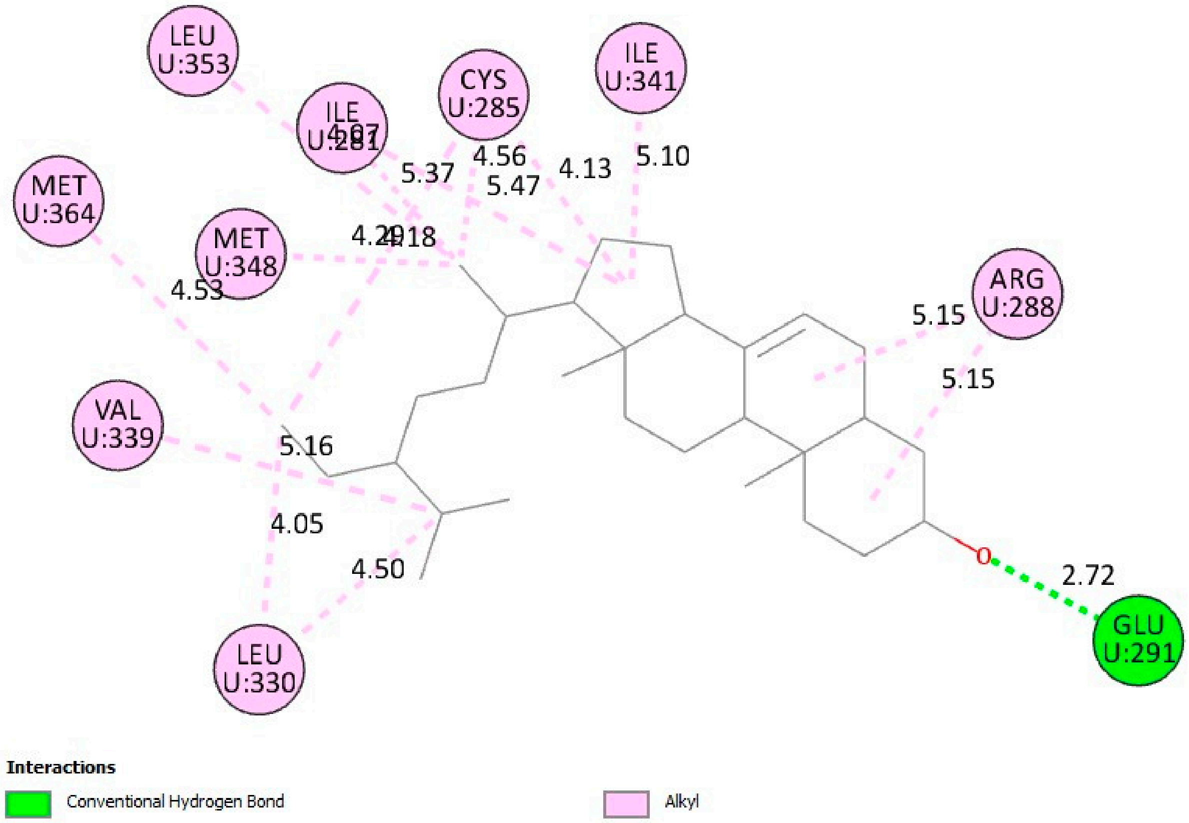

Supplementary Figure S10.

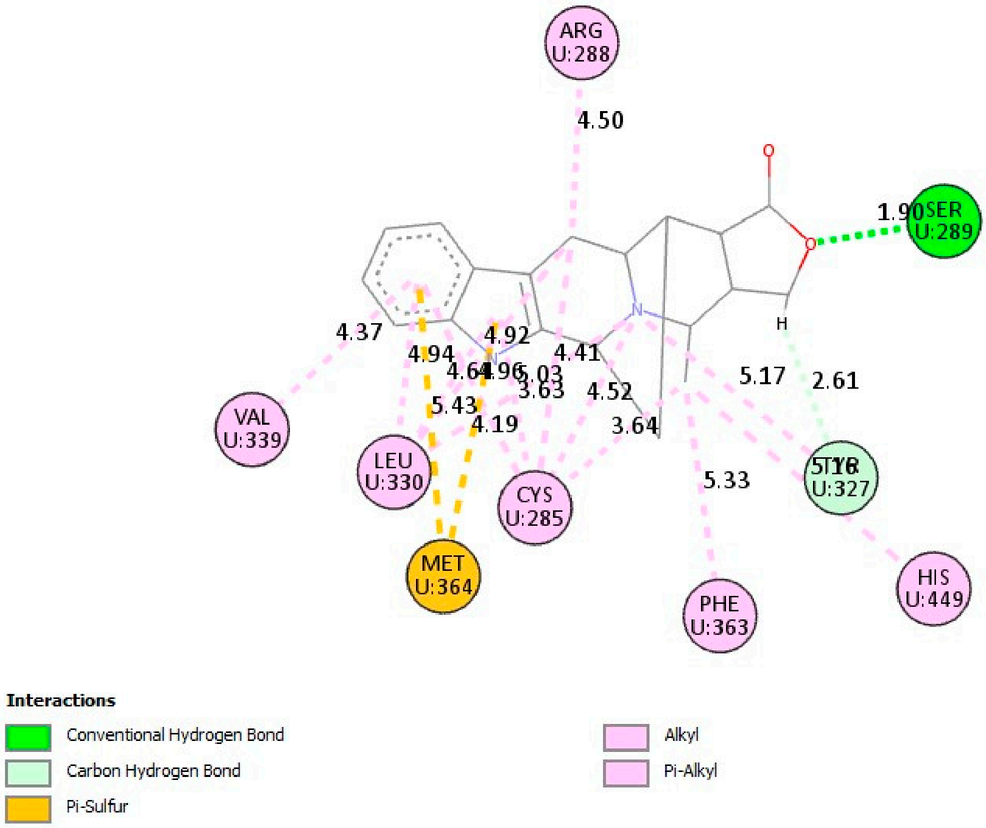

Supplementary Figure S11.

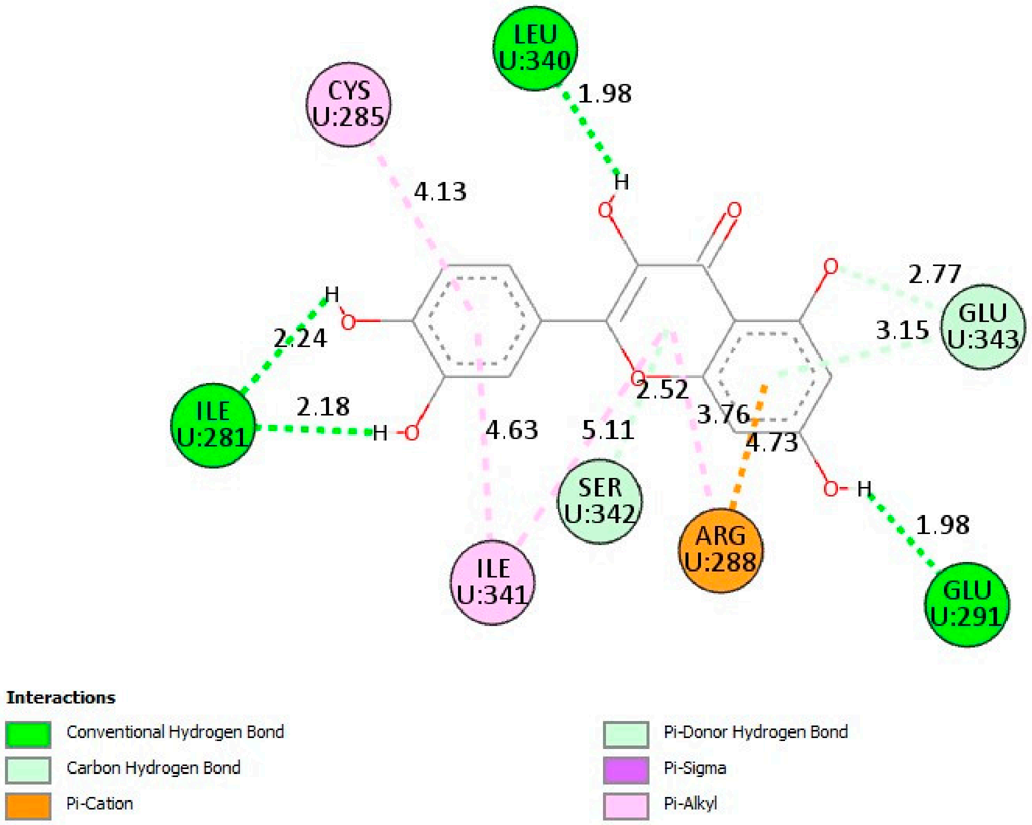

Supplementary Figure S12.

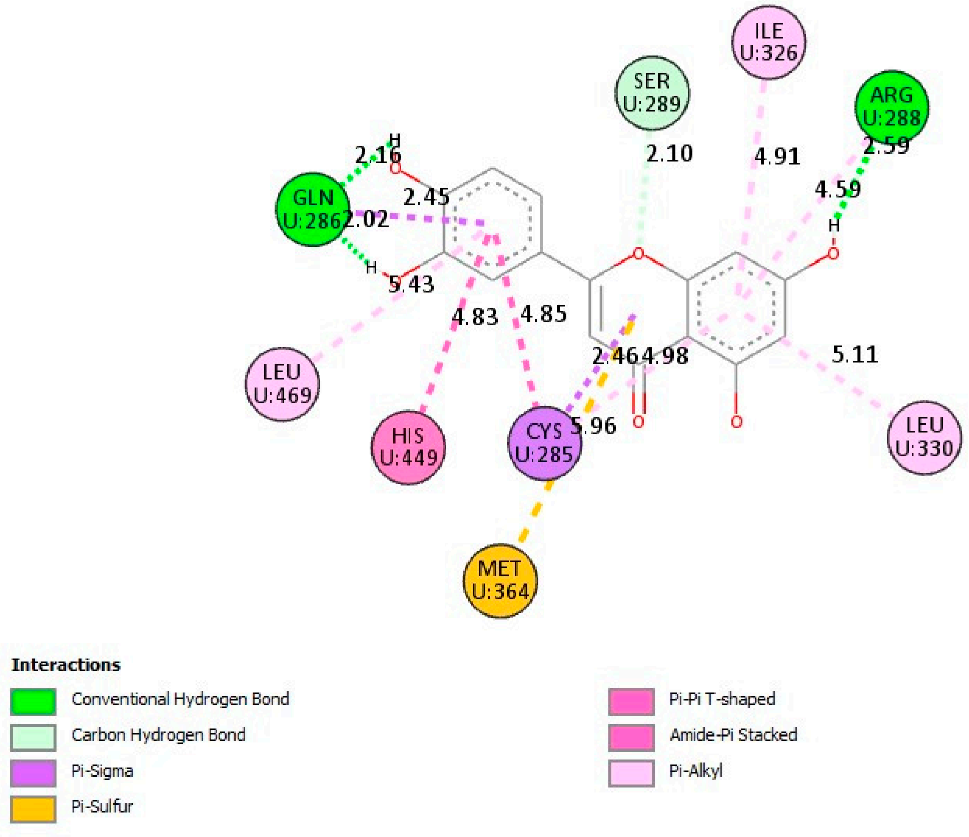

Supplementary Figure S13.

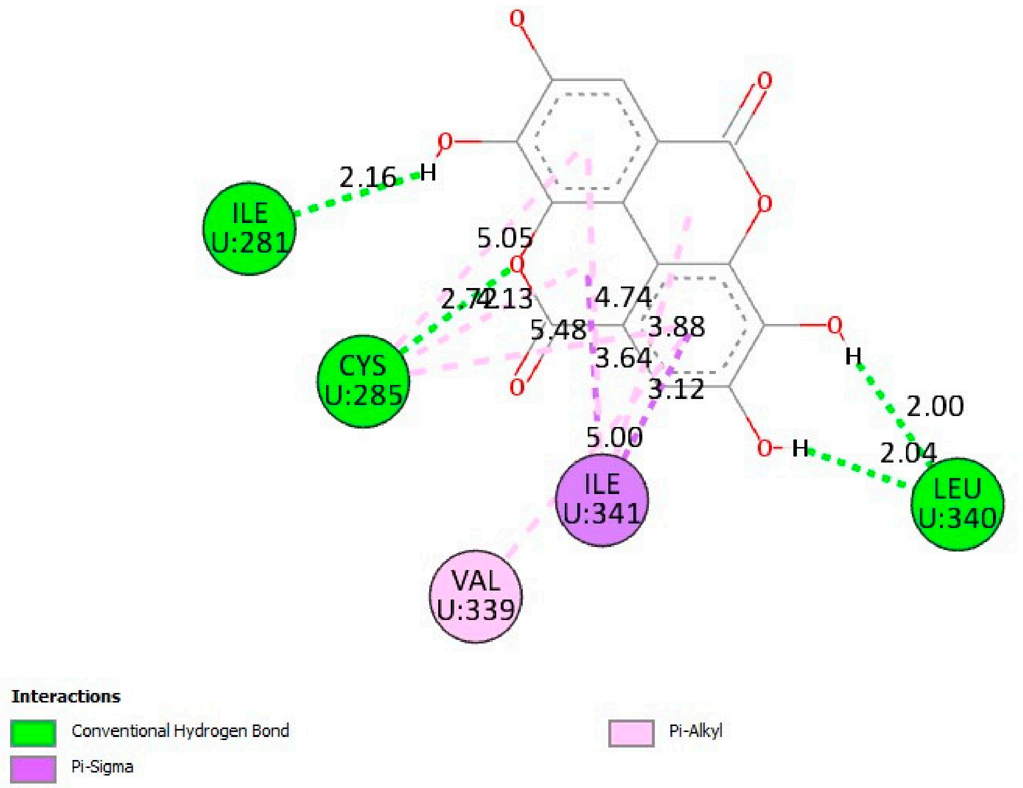

Supplementary Figure S14.

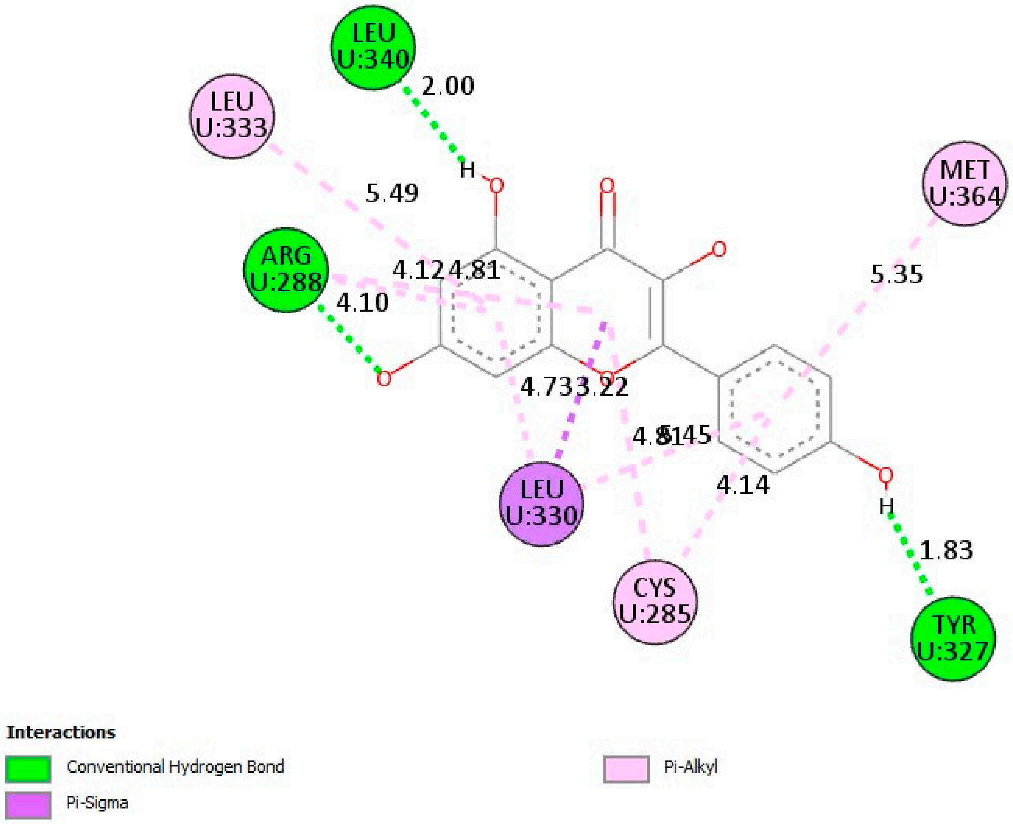

Supplementary Figure S15.

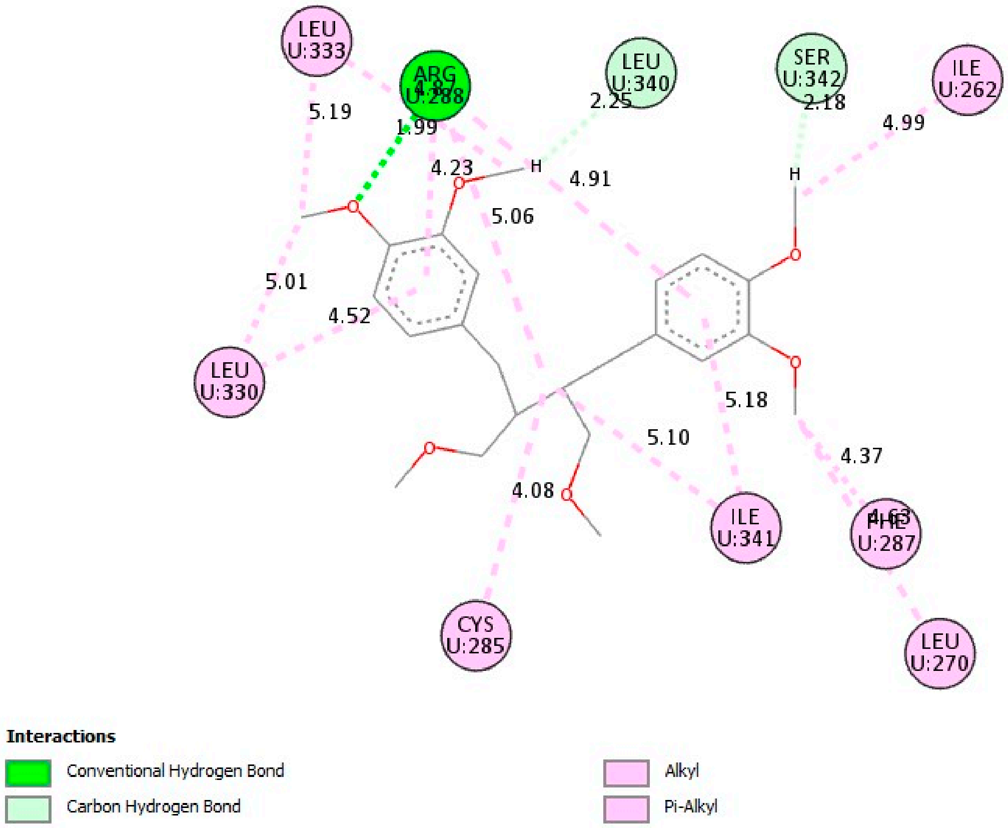

Supplementary Figure S16.

Supplementary Table S1.

Symbol	Uniprot name	Database
FTO	FTO_HUMAN	NCBI
BDNF	BDNF_HUMAN	NCBI
MC4R	MC4R_HUMAN	NCBI
ESR1	ESR1_HUMAN	NCBI
TLR4	TLR4_HUMAN	NCBI
NTRK2	NTRK2_HUMAN	NCBI
PPARG	PPARG_HUMAN	NCBI
WWOX	WWOX_HUMAN	NCBI
ENPP1	ENPP1_HUMAN	NCBI
ARG1	ARG1_HUMAN	NCBI
HMOX1	HMOX1_HUMAN	NCBI
TMEM18	TMEM18_HUMAN	NCBI
ADRB2	ADRB2_HUMAN	NCBI
FAS	TNR6_HUMAN	NCBI
LRRK2	LRRK2_HUMAN	NCBI
GHRL	GHRL_HUMAN	NCBI
IL1RN	IL1RA_HUMAN	NCBI
MGMT	MGMT_HUMAN	NCBI
UGT1A1	UD11_HUMAN	NCBI
DCC	DCC_HUMAN	NCBI
LPL	LIPL_HUMAN	NCBI
CNTNAP2	CNTP2_HUMAN	NCBI
LEPR	LEPR_HUMAN	NCBI
BMP2	BMP2_HUMAN	NCBI
TWIST1	TWST1_HUMAN	NCBI
SLC8A1	NAC1_HUMAN	NCBI
INHBB	INHBB_HUMAN	NCBI
CREBBP	CBP_HUMAN	NCBI
PTPN22	PTN22_HUMAN	NCBI
MTNR1A	MTR1A_HUMAN	NCBI
CYP2E1	CP2E1_HUMAN	NCBI
ASAH1	ASAH1_HUMAN	NCBI
DICER1	DICER_HUMAN	NCBI
APOA5	APOA5_HUMAN	NCBI
FAT1	FAT1_HUMAN	NCBI
OLFM4	OLFM4_HUMAN	NCBI

PNPLA3	PLPL3_HUMAN	NCBI
ERBB3	ERBB3_HUMAN	NCBI
CCR2	CCR2_HUMAN	NCBI
TIMP2	TIMP2_HUMAN	NCBI
TSC2	TSC2_HUMAN	NCBI
KLRK1	NKG2D_HUMAN	NCBI
ADRB3	ADRB3_HUMAN	NCBI
CBS	CBS_HUMAN	NCBI
SLC4A1	B3AT_HUMAN	NCBI
ETS1	ETS1_HUMAN	NCBI
LPP	LPP_HUMAN	NCBI
ABO	BGAT_HUMAN	NCBI
NOX4	NOX4_HUMAN	NCBI
RPS6KB1	KS6B1_HUMAN	NCBI
CDH2	CADH2_HUMAN	NCBI
MYB	MYB_HUMAN	NCBI
POMC	COLI_HUMAN	NCBI
LIPC	LIPC_HUMAN	NCBI
SLC28A3	S28A3_HUMAN	NCBI
DRD1	DRD1_HUMAN	NCBI
SPINK1	ISK1_HUMAN	NCBI
ZEB2	ZEB2_HUMAN	NCBI
RYR2	RYR2_HUMAN	NCBI
BAK1	BAK_HUMAN	NCBI
CSF1	CSF1_HUMAN	NCBI
UBE3A	UBE3A_HUMAN	NCBI
TCF4	ITF2_HUMAN	NCBI
GCH1	GCH1_HUMAN	NCBI
SERPING1	IC1_HUMAN	NCBI
SGK1	SGK1_HUMAN	NCBI
RARB	RARB_HUMAN	NCBI
CD46	MCP_HUMAN	NCBI
NRXN3	NRX3B_HUMAN	NCBI
PRF1	PERF_HUMAN	NCBI
KCNMA1	KCMA1_HUMAN	NCBI
TGFA	TGFA_HUMAN	NCBI
PDE4D	PDE4D_HUMAN	NCBI
XPA	XPA_HUMAN	NCBI
COL4A1	CO4A1_HUMAN	NCBI

NEGR1	NEGR1_HUMAN	NCBI
NPC1	NPC1_HUMAN	NCBI
CADM1	CADM1_HUMAN	NCBI
SLC30A8	ZNT8_HUMAN	NCBI
EDNRA	EDNRA_HUMAN	NCBI
MTNR1B	MTR1B_HUMAN	NCBI
UBE2E3	UB2E3_HUMAN	NCBI
CHRNA3	ACHA3_HUMAN	NCBI
PRKD1	KPCD1_HUMAN	NCBI
SEMA3A	SEM3A_HUMAN	NCBI
ITPR1	ITPR1_HUMAN	NCBI
KCNJ2	KCNJ2_HUMAN	NCBI
CCND2	CCND2_HUMAN	NCBI
PAX5	PAX5_HUMAN	NCBI
UGT2B7	UD2B7_HUMAN	NCBI
SCNN1A	SCNNA_HUMAN	NCBI
ACKR1	ACKR1_HUMAN	NCBI
DLC1	RHG07_HUMAN	NCBI
CTSS	CATS_HUMAN	NCBI
CHRM3	ACM3_HUMAN	NCBI
IFI16	IF16_HUMAN	NCBI
SERPINA12	SPA12_HUMAN	NCBI
UCP3	UCP3_HUMAN	NCBI
RTN4	RTN4_HUMAN	NCBI
FUT2	FUT2_HUMAN	NCBI
NOTCH4	NOTC4_HUMAN	NCBI
XDH	XDH_HUMAN	NCBI
ZNF804A	Z804A_HUMAN	NCBI
RASAL2	NGAP_HUMAN	NCBI
ICOS	ICOS_HUMAN	NCBI
ADCYAP1	PACA_HUMAN	NCBI
MRPS22	RT22_HUMAN	NCBI
CCR3	CCR3_HUMAN	NCBI
UGT1A9	UD19_HUMAN	NCBI
CD151	CD151_HUMAN	NCBI
USF1	USF1_HUMAN	NCBI
FCER1A	FCERA_HUMAN	NCBI
BICD1	BICD1_HUMAN	NCBI
DAOA	DAOA_HUMAN	NCBI

MLN	MOTI_HUMAN	NCBI
MAX	MAX_HUMAN	NCBI
E2F3	E2F3_HUMAN	NCBI
ITGBL1	ITGBL_HUMAN	NCBI
PRKG1	KGP1_HUMAN	NCBI
ILF3	ILF3_HUMAN	NCBI
PROX1	PROX1_HUMAN	NCBI
MACROD2	MACD2_HUMAN	NCBI
CDKAL1	CDKAL_HUMAN	NCBI
CPT1A	CPT1A_HUMAN	NCBI
HDAC9	HDAC9_HUMAN	NCBI
SOX11	SOX11_HUMAN	NCBI
TBX5	TBX5_HUMAN	NCBI
S100P	S100P_HUMAN	NCBI
CAMK2A	KCC2A_HUMAN	NCBI
AIM2	AIM2_HUMAN	NCBI
TREX1	TREX1_HUMAN	NCBI
LIPG	LIPE_HUMAN	NCBI
IL10RA	I10R1_HUMAN	NCBI
PIK3C3	PK3C3_HUMAN	NCBI
FOXE1	FOXE1_HUMAN	NCBI
ECE1	ECE1_HUMAN	NCBI
RICTOR	RICTR_HUMAN	NCBI
DCLK1	DCLK1_HUMAN	NCBI
POLD1	DPOD1_HUMAN	NCBI
PKP2	PKP2_HUMAN	NCBI
EXT1	EXT1_HUMAN	NCBI
GRIA1	GRIA1_HUMAN	NCBI
GTF2B	TF2B_HUMAN	NCBI
THBS2	TSP2_HUMAN	NCBI
NOS1AP	CAPON_HUMAN	NCBI
NIPBL	NIPBL_HUMAN	NCBI
ADORA3	AA3R_HUMAN	NCBI
PRKCH	KPCL_HUMAN	NCBI
NOX5	NOX5_HUMAN	NCBI
NR0B2	NR0B2_HUMAN	NCBI
NCAM2	NCAM2_HUMAN	NCBI
FBP1	F16P1_HUMAN	NCBI
NRF1	NRF1_HUMAN	NCBI

RNF2	RING2_HUMAN	NCBI
UGT1A6	UD16_HUMAN	NCBI
ECT2	ECT2_HUMAN	NCBI
KCTD15	KCD15_HUMAN	NCBI
HAS2	HYAS2_HUMAN	NCBI
KCNN3	KCNN3_HUMAN	NCBI
GRK5	GRK5_HUMAN	NCBI
TNKS	TNKS1_HUMAN	NCBI
SMYD3	SMYD3_HUMAN	NCBI
NUP153	NU153_HUMAN	NCBI
MAF	MAF_HUMAN	NCBI
FSTL1	FSTL1_HUMAN	NCBI
SPTB	SPTB1_HUMAN	NCBI
GRIK2	GRIK2_HUMAN	NCBI
SOCS2	SOCS2_HUMAN	NCBI
SYT1	SYT1_HUMAN	NCBI
WNT4	WNT4_HUMAN	NCBI
ITPR3	ITPR3_HUMAN	NCBI
LRP8	LRP8_HUMAN	NCBI
PCDH9	PCDH9_HUMAN	NCBI
FUT8	FUT8_HUMAN	NCBI
CD83	CD83_HUMAN	NCBI
TRIB1	TRIB1_HUMAN	NCBI
PTPRD	PTPRD_HUMAN	NCBI
KLK4	KLK4_HUMAN	NCBI
HDAC7	HDAC7_HUMAN	NCBI
MACROH2A1	H2AY_HUMAN	NCBI
PPARGC1B	PRGC2_HUMAN	NCBI
MAFB	MAFB_HUMAN	NCBI
UGT1A7	UD17_HUMAN	NCBI
BACH2	BACH2_HUMAN	NCBI
AIF1	AIF1_HUMAN	NCBI
NCR3	NCTR3_HUMAN	NCBI
PITPNB	PIPNB_HUMAN	NCBI
DOCK8	DOCK8_HUMAN	NCBI
CARD9	CARD9_HUMAN	NCBI
KCNB1	KCNB1_HUMAN	NCBI
FOXF1	FOXF1_HUMAN	NCBI
CSMD1	CSMD1_HUMAN	NCBI

CTCFL	CTCFL_HUMAN	NCBI
ENAH	ENAH_HUMAN	NCBI
TBXAS1	THAS_HUMAN	NCBI
SNRPN	RSMN_HUMAN	NCBI
CARTPT	CART_HUMAN	NCBI
PLOD2	PLOD2_HUMAN	NCBI
ITGB7	ITB7_HUMAN	NCBI
DLG5	DLG5_HUMAN	NCBI
BCAM	BCAM_HUMAN	NCBI
IREB2	IREB2_HUMAN	NCBI
ABCC9	ABCC9_HUMAN	NCBI
KLKB1	KLKB1_HUMAN	NCBI
SH2B1	SH2B1_HUMAN	NCBI
ADCY5	ADCY5_HUMAN	NCBI
SOX6	SOX6_HUMAN	NCBI
ACD	ACD_HUMAN	NCBI
HTATIP2	HTAI2_HUMAN	NCBI
RNASE1	RNAS1_HUMAN	NCBI
IFNGR2	INGR2_HUMAN	NCBI
RAB6A	RAB6A_HUMAN	NCBI
RBFOX1	RFOX1_HUMAN	NCBI
EFNA1	EFNA1_HUMAN	NCBI
CD109	CD109_HUMAN	NCBI
MAML2	MAML2_HUMAN	NCBI
OSMR	OSMR_HUMAN	NCBI
PIP4K2A	PI42A_HUMAN	NCBI
HR	HAIR_HUMAN	NCBI
DBI	ACBP_HUMAN	NCBI
TRA2B	TRA2B_HUMAN	NCBI
PRMT6	ANM6_HUMAN	NCBI
AGRP	AGRP_HUMAN	NCBI
RAD51B	RA51B_HUMAN	NCBI
NECTIN2	NECT2_HUMAN	NCBI
FBN2	FBN2_HUMAN	NCBI
SS18	SSXT_HUMAN	NCBI
CITED2	CITE2_HUMAN	NCBI
UGT1A4	UD14_HUMAN	NCBI
EBF1	COE1_HUMAN	NCBI
CD93	C1QR1_HUMAN	NCBI

FRZB	SFRP3_HUMAN	NCBI
ADH5	ADHX_HUMAN	NCBI
MYO15A	MYO15_HUMAN	NCBI
KCNE2	KCNE2_HUMAN	NCBI
FMOD	FMOD_HUMAN	NCBI
GRIN3A	NMD3A_HUMAN	NCBI
RTN4R	RTN4R_HUMAN	NCBI
TRPC4	TRPC4_HUMAN	NCBI
NELL1	NELL1_HUMAN	NCBI
ZNF217	ZN217_HUMAN	NCBI
KPNA3	IMA4_HUMAN	NCBI
ETV5	ETV5_HUMAN	NCBI
SH3GL2	SH3G2_HUMAN	NCBI
CLEC16A	CL16A_HUMAN	NCBI
MSRA	MSRA_HUMAN	NCBI
KDM4C	KDM4C_HUMAN	NCBI
STIM2	STIM2_HUMAN	NCBI
ANAPC1	APC1_HUMAN	NCBI
ATP2A1	AT2A1_HUMAN	NCBI
EHF	EHF_HUMAN	NCBI
CACNB2	CACB2_HUMAN	NCBI
DLG2	DLG2_HUMAN	NCBI
SLC19A3	S19A3_HUMAN	NCBI
ALKBH5	ALKB5_HUMAN	NCBI
ELAVL4	ELAV4_HUMAN	NCBI
DSC3	DSC3_HUMAN	NCBI
KIF6	KIF6_HUMAN	NCBI
PRM1	HSP1_HUMAN	NCBI
NAP1L1	NP1L1_HUMAN	NCBI
AUTS2	AUTS2_HUMAN	NCBI
MBOAT7	MBOA7_HUMAN	NCBI
PVALB	PRVA_HUMAN	NCBI
PAK5	PAK5_HUMAN	NCBI
CACNA2D1	CA2D1_HUMAN	NCBI
UGT1A10	UD110_HUMAN	NCBI
SP110	SP110_HUMAN	NCBI
RFC5	RFC5_HUMAN	NCBI
MAGI2	MAGI2_HUMAN	NCBI
AATF	AATF_HUMAN	NCBI

UGT1A8	UD18_HUMAN	NCBI
HPSE2	HPSE2_HUMAN	NCBI
PMEPA1	PMEPA_HUMAN	NCBI
CA8	CAH8_HUMAN	NCBI
TTC28	TTC28_HUMAN	NCBI
ACKR2	ACKR2_HUMAN	NCBI
CTNNA3	CTNA3_HUMAN	NCBI
KIDINS220	KDIS_HUMAN	NCBI
RAMP1	RAMP1_HUMAN	NCBI
PARD6A	PAR6A_HUMAN	NCBI
PCSK2	NEC2_HUMAN	NCBI
GPC5	GPC5_HUMAN	NCBI
DYRK1B	DYR1B_HUMAN	NCBI
NACC1	NACC1_HUMAN	NCBI
HPS1	HPS1_HUMAN	NCBI
RFC3	RFC3_HUMAN	NCBI
CCN5	CCN5_HUMAN	NCBI
TRAPPC9	TPPC9_HUMAN	NCBI
RBBP6	RBBP6_HUMAN	NCBI
CELF2	CELF2_HUMAN	NCBI
ADGRV1	AGRV1_HUMAN	NCBI
TANK	TANK_HUMAN	NCBI
HOXB5	HXB5_HUMAN	NCBI
UGT1A3	UD13_HUMAN	NCBI
SLC29A3	S29A3_HUMAN	NCBI
POU3F2	PO3F2_HUMAN	NCBI
GABRB1	GBRB1_HUMAN	NCBI
HLX	HLX_HUMAN	NCBI
SORBS1	SRBS1_HUMAN	NCBI
RHOT1	MIRO1_HUMAN	NCBI
NRG3	NRG3_HUMAN	NCBI
CHL1	NCHL1_HUMAN	NCBI
SHANK2	SHAN2_HUMAN	NCBI
SGCD	SGCD_HUMAN	NCBI
GREB1	GREB1_HUMAN	NCBI
CPEB4	CPEB4_HUMAN	NCBI
TOMM20	TOM20_HUMAN	NCBI
CHN2	CHIO_HUMAN	NCBI
EMX2	EMX2_HUMAN	NCBI

UNC5C	UNC5C_HUMAN	NCBI
ARPC2	ARPC2_HUMAN	NCBI
CD53	CD53_HUMAN	NCBI
PAK6	PAK6_HUMAN	NCBI
SDC3	SDC3_HUMAN	NCBI
SEMA5A	SEM5A_HUMAN	NCBI
MYO10	MYO10_HUMAN	NCBI
YTHDC1	YTDC1_HUMAN	NCBI
MATK	MATK_HUMAN	NCBI
CDH4	CADH4_HUMAN	NCBI
SLC22A11	S22AB_HUMAN	NCBI
PSAT1	SERC_HUMAN	NCBI
PKP4	PKP4_HUMAN	NCBI
ASIC2	ASIC2_HUMAN	NCBI
MYO1B	MYO1B_HUMAN	NCBI
DNAJC10	DJC10_HUMAN	NCBI
DPP10	DPP10_HUMAN	NCBI
OTUD7B	OTU7B_HUMAN	NCBI
GTF2E1	T2EA_HUMAN	NCBI
GALR1	GALR1_HUMAN	NCBI
KCNN2	KCNN2_HUMAN	NCBI
SRSF10	SRS10_HUMAN	NCBI
ICA1	ICA69_HUMAN	NCBI
FGFRL1	FGRL1_HUMAN	NCBI
PACS1	PACS1_HUMAN	NCBI
KCNV2	KCNV2_HUMAN	NCBI
PMS1	PMS1_HUMAN	NCBI
SLC6A5	SC6A5_HUMAN	NCBI
SSTR4	SSR4_HUMAN	NCBI
ZPR1	ZPR1_HUMAN	NCBI
LHFPL3	LHPL3_HUMAN	NCBI
SCG5	7B2_HUMAN	NCBI
SRP14	SRP14_HUMAN	NCBI
KMO	KMO_HUMAN	NCBI
FARS2	SYFM_HUMAN	NCBI
PCBD1	PHS_HUMAN	NCBI
FAIM2	LFG2_HUMAN	NCBI
IPMK	IPMK_HUMAN	NCBI
ARHGAP24	RHG24_HUMAN	NCBI

RSU1	RSU1_HUMAN	NCBI
DMRT1	DMRT1_HUMAN	NCBI
MNDA	MNDA_HUMAN	NCBI
PARP4	PARP4_HUMAN	NCBI
PKD2L1	PK2L1_HUMAN	NCBI
RLN3	REL3_HUMAN	NCBI
ANO10	ANO10_HUMAN	NCBI
JDP2	JDP2_HUMAN	NCBI
DSCAM	DSCAM_HUMAN	NCBI
PZP	PZP_HUMAN	NCBI
TMOD1	TMOD1_HUMAN	NCBI
CPSF2	CPSF2_HUMAN	NCBI
DPYSL5	DPYL5_HUMAN	NCBI
HMBOX1	HMBX1_HUMAN	NCBI
PALM2AKAP2	AKAP2_HUMAN	NCBI
ATP12A	AT12A_HUMAN	NCBI
RP1L1	RP1L1_HUMAN	NCBI
CAMK1D	KCC1D_HUMAN	NCBI
PTPRN2	PTPR2_HUMAN	NCBI
PATJ	INADL_HUMAN	NCBI
RAMP3	RAMP3_HUMAN	NCBI
SLC35A1	S35A1_HUMAN	NCBI
DYNC1I1	DC1I1_HUMAN	NCBI
KIF7	KIF7_HUMAN	NCBI
RASSF10	RASFA_HUMAN	NCBI
KCNK5	KCNK5_HUMAN	NCBI
ANKS1B	ANS1B_HUMAN	NCBI
CLTB	CLCB_HUMAN	NCBI
PRDM5	PRDM5_HUMAN	NCBI
CHST11	CHSTB_HUMAN	NCBI
CELA1	CELA1_HUMAN	NCBI
CPLX2	CPLX2_HUMAN	NCBI
MYO3A	MYO3A_HUMAN	NCBI
TLL1	TLL1_HUMAN	NCBI
KDM8	KDM8_HUMAN	NCBI
SQOR	SQOR_HUMAN	NCBI
MGAT1	MGAT1_HUMAN	NCBI
RGS7	RGS7_HUMAN	NCBI
DGKG	DGKG_HUMAN	NCBI

MPP7	MPP7_HUMAN	NCBI
TACC2	TACC2_HUMAN	NCBI
ALPK1	ALPK1_HUMAN	NCBI
NINJ1	NINJ1_HUMAN	NCBI
DLGAP1	DLGP1_HUMAN	NCBI
ISG20	ISG20_HUMAN	NCBI
TBL2	TBL2_HUMAN	NCBI
HOXB3	HXB3_HUMAN	NCBI
NMNAT2	NMNA2_HUMAN	NCBI
KIF1C	KIF1C_HUMAN	NCBI
LHPP	LHPP_HUMAN	NCBI
HPD	HPPD_HUMAN	NCBI
SEPTIN11	SEP11_HUMAN	NCBI
CMYA5	CMYA5_HUMAN	NCBI
NFE2L3	NF2L3_HUMAN	NCBI
BRAT1	BRAT1_HUMAN	NCBI
ARHGEF3	ARHG3_HUMAN	NCBI
GRID2	GRID2_HUMAN	NCBI
WHRN	WHRN_HUMAN	NCBI
SUPT3H	SUPT3_HUMAN	NCBI
SDCCAG8	SDCG8_HUMAN	NCBI
MPRIP	MPRIP_HUMAN	NCBI
UBR2	UBR2_HUMAN	NCBI
GPC6	GPC6_HUMAN	NCBI
MAPK15	MK15_HUMAN	NCBI
UNC13A	UN13A_HUMAN	NCBI
PDCL	PHLP_HUMAN	NCBI
CEP72	CEP72_HUMAN	NCBI
CEP152	CE152_HUMAN	NCBI
ORC3	ORC3_HUMAN	NCBI
BAIAP2L1	BI2L1_HUMAN	NCBI
ACKR4	ACKR4_HUMAN	NCBI
ASTN2	ASTN2_HUMAN	NCBI
PLA2G2D	PA2GD_HUMAN	NCBI
MAGI3	MAGI3_HUMAN	NCBI
TUB	TUB_HUMAN	NCBI
ELOVL6	ELOV6_HUMAN	NCBI
ANO3	ANO3_HUMAN	NCBI
PCDH7	PCDH7_HUMAN	NCBI

FOXL1	FOXL1_HUMAN	NCBI
CDHR3	CDHR3_HUMAN	NCBI
ANKS1A	ANS1A_HUMAN	NCBI
NREP	NREP_HUMAN	NCBI
MAEA	MAEA_HUMAN	NCBI
ECI2	ECI2_HUMAN	NCBI
TSBP1	TSBP1_HUMAN	NCBI
PPP1R12B	MYPT2_HUMAN	NCBI
CPVL	CPVL_HUMAN	NCBI
PUM3	PUM3_HUMAN	NCBI
TICRR	TICRR_HUMAN	NCBI
PHIP	PHIP_HUMAN	NCBI
EVA1A	EVA1A_HUMAN	NCBI
LRATD2	LRAT2_HUMAN	NCBI
MSRB2	MSRB2_HUMAN	NCBI
GMDS	GMDS_HUMAN	NCBI
NCEH1	NCEH1_HUMAN	NCBI
HIGD1A	HIG1A_HUMAN	NCBI
STON2	STON2_HUMAN	NCBI
GUCY1A2	GCYA2_HUMAN	NCBI
CUL9	CUL9_HUMAN	NCBI
RARS2	SYRM_HUMAN	NCBI
CEP120	CE120_HUMAN	NCBI
PDZD2	PDZD2_HUMAN	NCBI
COLEC12	COL12_HUMAN	NCBI
NIBAN1	NIBA1_HUMAN	NCBI
KCNIP4	KCIP4_HUMAN	NCBI
MUC15	MUC15_HUMAN	NCBI
FHOD3	FHOD3_HUMAN	NCBI
KIRREL1	KIRR1_HUMAN	NCBI
LRRC7	LRRC7_HUMAN	NCBI
ADAMTS14	ATS14_HUMAN	NCBI
WDR11	WDR11_HUMAN	NCBI
CC2D2A	C2D2A_HUMAN	NCBI
CNTNAP1	CNTP1_HUMAN	NCBI
PFDN4	PFD4_HUMAN	NCBI
BCAS3	BCAS3_HUMAN	NCBI
MXD3	MAD3_HUMAN	NCBI
NDUFA8	NDUA8_HUMAN	NCBI

NXT1	NXT1_HUMAN	NCBI
NTM	NTRI_HUMAN	NCBI
EPHA6	EPHA6_HUMAN	NCBI
AP4B1	AP4B1_HUMAN	NCBI
TOM1L1	TM1L1_HUMAN	NCBI
MDFIC	MDFIC_HUMAN	NCBI
RAB17	RAB17_HUMAN	NCBI
SIPA1L1	SI1L1_HUMAN	NCBI
BICC1	BICC1_HUMAN	NCBI
PTDSS1	PTSS1_HUMAN	NCBI
COQ4	COQ4_HUMAN	NCBI
ABLIM1	ABLM1_HUMAN	NCBI
PIP5K1B	PI51B_HUMAN	NCBI
POU3F1	PO3F1_HUMAN	NCBI
PCF11	PCF11_HUMAN	NCBI
GPRC5B	GPC5B_HUMAN	NCBI
SPECC1	CYTSB_HUMAN	NCBI
SRRM4	SRRM4_HUMAN	NCBI
LEMD2	LEMD2_HUMAN	NCBI
ANO2	ANO2_HUMAN	NCBI
SEC16B	SC16B_HUMAN	NCBI
USP6NL	US6NL_HUMAN	NCBI
TMED9	TMED9_HUMAN	NCBI
GPSM3	GPSM3_HUMAN	NCBI
BDP1	BDP1_HUMAN	NCBI
FARP1	FARP1_HUMAN	NCBI
RAB28	RAB28_HUMAN	NCBI
GLRX5	GLRX5_HUMAN	NCBI
SLMAP	SLMAP_HUMAN	NCBI
MDGA2	MDGA2_HUMAN	NCBI
SLCO3A1	SO3A1_HUMAN	NCBI
MBNL2	MBNL2_HUMAN	NCBI
HECW1	HECW1_HUMAN	NCBI
RABL3	RABL3_HUMAN	NCBI
SPAG17	SPG17_HUMAN	NCBI
RIDA	RIDA_HUMAN	NCBI
EPSTI1	ESIP1_HUMAN	NCBI
AEBP2	AEBP2_HUMAN	NCBI
NAV2	NAV2_HUMAN	NCBI

RHPN2	RHPN2_HUMAN	NCBI
FSIP1	FSIP1_HUMAN	NCBI
TMEM132D	T132D_HUMAN	NCBI
VGLL3	VGLL3_HUMAN	NCBI
RIN2	RIN2_HUMAN	NCBI
PPM1H	PPM1H_HUMAN	NCBI
SUGP1	SUGP1_HUMAN	NCBI
SPTLC3	SPTC3_HUMAN	NCBI
ZC3H4	ZC3H4_HUMAN	NCBI
FGGY	FGGY_HUMAN	NCBI
CDK5R2	CD5R2_HUMAN	NCBI
ADSS2	PURA2_HUMAN	NCBI
PKN3	PKN3_HUMAN	NCBI
FBXL7	FBXL7_HUMAN	NCBI
UBXN7	UBXN7_HUMAN	NCBI
COX15	COX15_HUMAN	NCBI
NXPH1	NXPH1_HUMAN	NCBI
ABHD2	ABHD2_HUMAN	NCBI
DCP1B	DCP1B_HUMAN	NCBI
TUBGCP6	GCP6_HUMAN	NCBI
ADAMTSL1	ATL1_HUMAN	NCBI
OLFML3	OLFL3_HUMAN	NCBI
LINGO2	LIGO2_HUMAN	NCBI
LNPK	LNP_HUMAN	NCBI
AATK	LMTK1_HUMAN	NCBI
CACTIN	CATIN_HUMAN	NCBI
ARHGAP11A	RHGBA_HUMAN	NCBI
FICD	FICD_HUMAN	NCBI
CWC27	CWC27_HUMAN	NCBI
SLIT1	SLIT1_HUMAN	NCBI
SCRN1	SCRN1_HUMAN	NCBI
BCDIN3D	BN3D2_HUMAN	NCBI
CFAP418	CF418_HUMAN	NCBI
AOAH	AOAH_HUMAN	NCBI
PKNOX2	PKNX2_HUMAN	NCBI
EDEM3	EDEM3_HUMAN	NCBI
TGFBRAP1	TGFA1_HUMAN	NCBI
TM9SF2	TM9S2_HUMAN	NCBI
KIAA1217	SKT_HUMAN	NCBI

SPAG16	SPG16_HUMAN	NCBI
SNRK	SNRK_HUMAN	NCBI
PELI2	PELI2_HUMAN	NCBI
OTUD1	OTUD1_HUMAN	NCBI
TENM4	TEN4_HUMAN	NCBI
PLEKHG6	PKHG6_HUMAN	NCBI
GPAT3	GPAT3_HUMAN	NCBI
TMC2	TMC2_HUMAN	NCBI
CEP112	CE112_HUMAN	NCBI
UGT1A5	UD15_HUMAN	NCBI
ASPRV1	APRV1_HUMAN	NCBI
MRPL48	RM48_HUMAN	NCBI
LAYN	LAYN_HUMAN	NCBI
CNTN5	CNTN5_HUMAN	NCBI
SORCS2	SORC2_HUMAN	NCBI
TFPT	TFPT_HUMAN	NCBI
DCTD	DCTD_HUMAN	NCBI
SLC39A11	S39AB_HUMAN	NCBI
TTC8	TTC8_HUMAN	NCBI
LRFN2	LRFN2_HUMAN	NCBI
TMEM45B	TM45B_HUMAN	NCBI
NAV1	NAV1_HUMAN	NCBI
GALNT13	GLT13_HUMAN	NCBI
PSD3	PSD3_HUMAN	NCBI
BET1	BET1_HUMAN	NCBI
CEP85	CEP85_HUMAN	NCBI
MTUS2	MTUS2_HUMAN	NCBI
AFAP1L1	AF1L1_HUMAN	NCBI
DHX57	DHX57_HUMAN	NCBI
ACAD8	ACAD8_HUMAN	NCBI
ACMSD	ACMSD_HUMAN	NCBI
CHST8	CHST8_HUMAN	NCBI
BRI3	BRI3_HUMAN	NCBI
KLHL1	KLHL1_HUMAN	NCBI
TUBD1	TBD_HUMAN	NCBI
MRPS24	RT24_HUMAN	NCBI
AGMO	ALKMO_HUMAN	NCBI
B3GAT2	B3GA2_HUMAN	NCBI
CLDN23	CLD23_HUMAN	NCBI

PANK4	PANK4_HUMAN	NCBI
ZBTB46	ZBT46_HUMAN	NCBI
NALF1	NALF1_HUMAN	NCBI
MALRD1	MALR1_HUMAN	NCBI
RFTN1	RFTN1_HUMAN	NCBI
UBXN11	UBX11_HUMAN	NCBI
OLFM1	NOE1_HUMAN	NCBI
TPRN	TPRN_HUMAN	NCBI
PNRC2	PNRC2_HUMAN	NCBI
DCHS2	PCD23_HUMAN	NCBI
MPHOSPH6	MPH6_HUMAN	NCBI
WDR59	WDR59_HUMAN	NCBI
SGCZ	SGCZ_HUMAN	NCBI
ASB3	ASB3_HUMAN	NCBI
ARAP2	ARAP2_HUMAN	NCBI
NAALADL2	NADL2_HUMAN	NCBI
SYT6	SYT6_HUMAN	NCBI
WDPCP	FRITZ_HUMAN	NCBI
SDK1	SDK1_HUMAN	NCBI
LRIF1	LRIF1_HUMAN	NCBI
NCKAP1L	NCKPL_HUMAN	NCBI
TSEN34	SEN34_HUMAN	NCBI
RADIL	RADIL_HUMAN	NCBI
ACTR3B	ARP3B_HUMAN	NCBI
ADAMTS19	ATS19_HUMAN	NCBI
MYO16	F8W883_HUMAN	NCBI
PTER	PTER_HUMAN	NCBI
PBX4	PBX4_HUMAN	NCBI
DEUP1	DEUP1_HUMAN	NCBI
LGSN	LGSN_HUMAN	NCBI
PHTF1	PHTF1_HUMAN	NCBI
PHACTR2	PHAR2_HUMAN	NCBI
IGSF11	IGS11_HUMAN	NCBI
NPM2	NPM2_HUMAN	NCBI
RASEF	RASEF_HUMAN	NCBI
PCDH18	PCD18_HUMAN	NCBI
SAMD4A	SMAG1_HUMAN	NCBI
DSTYK	DUSTY_HUMAN	NCBI
ZNF536	ZN536_HUMAN	NCBI

SLC22A23	S22AN_HUMAN	NCBI
PRTFDC1	PRDC1_HUMAN	NCBI
PLEKHG1	PKHG1_HUMAN	NCBI
ARHGEF10L	ARGAL_HUMAN	NCBI
POM121C	P121C_HUMAN	NCBI
ATAD2B	ATD2B_HUMAN	NCBI
MBOAT1	MBOA1_HUMAN	NCBI
HOXB-AS3	HAS3P_HUMAN	NCBI
B3GALT1	B3GT1_HUMAN	NCBI
INO80D	IN80D_HUMAN	NCBI
AFG1L	AFG1L_HUMAN	NCBI
TRABD2B	TIKI2_HUMAN	NCBI
TRAPPC12	TPC12_HUMAN	NCBI
COL23A1	CONA1_HUMAN	NCBI
PLGRKT	PLRKT_HUMAN	NCBI
TRUB2	TRUB2_HUMAN	NCBI
UBXN2A	UBX2A_HUMAN	NCBI
SAMD4B	SMAG2_HUMAN	NCBI
MYO3B	MYO3B_HUMAN	NCBI
TCFL5	TCFL5_HUMAN	NCBI
GTDC1	GTDC1_HUMAN	NCBI
CUTC	CUTC_HUMAN	NCBI
RSBN1	RSBN1_HUMAN	NCBI
INCA1	INCA1_HUMAN	NCBI
IGSF9B	TUTLB_HUMAN	NCBI
SPOCK3	TICN3_HUMAN	NCBI
MMD	PAQRB_HUMAN	NCBI
UMODL1	UROL1_HUMAN	NCBI
HSP90AB2P	H90B2_HUMAN	NCBI
HS3ST4	HS3S4_HUMAN	NCBI
TAFA2	TAFA2_HUMAN	NCBI
VWA5A	VMA5A_HUMAN	NCBI
INSC	INSC_HUMAN	NCBI
UBTD1	UBTD1_HUMAN	NCBI
CLYBL	CLYBL_HUMAN	NCBI
EEPD1	EEPD1_HUMAN	NCBI
CCDC91	CCD91_HUMAN	NCBI
AOPEP	AMPO_HUMAN	NCBI
SLC25A2	ORNT2_HUMAN	NCBI

DIRC1	DIRC1_HUMAN	NCBI
CCDC69	CCD69_HUMAN	NCBI
FOXD2	FOXD2_HUMAN	NCBI
MOSPD2	MSPD2_HUMAN	NCBI
MOV10L1	M10L1_HUMAN	NCBI
PYROXD2	PYRD2_HUMAN	NCBI
DUSP29	DUS29_HUMAN	NCBI
CEP19	CEP19_HUMAN	NCBI
PHTF2	PHTF2_HUMAN	NCBI
SNX29	SNX29_HUMAN	NCBI
MVB12B	MB12B_HUMAN	NCBI
SETD4	SETD4_HUMAN	NCBI
CRYL1	CRYL1_HUMAN	NCBI
ACTL8	ACTL8_HUMAN	NCBI
KCNK15	KCNKF_HUMAN	NCBI
MOXD1	MOXD1_HUMAN	NCBI
FAM53B	FA53B_HUMAN	NCBI
RASGEF1A	RGF1A_HUMAN	NCBI
FCSK	FCSK_HUMAN	NCBI
GRXCR1	GRCR1_HUMAN	NCBI
ADAMTS20	ATS20_HUMAN	NCBI
TTLL7	TTLL7_HUMAN	NCBI
CYLC2	CYLC2_HUMAN	NCBI
AK8	KAD8_HUMAN	NCBI
DENND3	DEND3_HUMAN	NCBI
PLPPR5	PLPR5_HUMAN	NCBI
ODAD2	ODAD2_HUMAN	NCBI
SPATA8	SPAT8_HUMAN	NCBI
SLC36A4	S36A4_HUMAN	NCBI
CSAD	CSAD_HUMAN	NCBI
METTL15	MET15_HUMAN	NCBI
RNFT1	RNFT1_HUMAN	NCBI
TMEM30B	CC50B_HUMAN	NCBI
RSBN1L	RSBNL_HUMAN	NCBI
CCDC33	CCD33_HUMAN	NCBI
KLHL29	KLH29_HUMAN	NCBI
BTBD8	K1107_HUMAN	NCBI
ARHGAP28	RHG28_HUMAN	NCBI
BTBD3	BTBD3_HUMAN	NCBI

NLRP8	NALP8_HUMAN	NCBI
PKHD1L1	PKHL1_HUMAN	NCBI
FAM110A	F110A_HUMAN	NCBI
ZNF385D	Z385D_HUMAN	NCBI
FERD3L	FER3L_HUMAN	NCBI
SLC46A3	S46A3_HUMAN	NCBI
C20orf85	CT085_HUMAN	NCBI
ALLC	ALLC_HUMAN	NCBI
KBTBD11	KBTBB_HUMAN	NCBI
DDX60L	DDX6L_HUMAN	NCBI
CABCOCO1	CBCO1_HUMAN	NCBI
ZPLD1	ZPLD1_HUMAN	NCBI
CRACR2B	EFC4A_HUMAN	NCBI
CCDC77	CCD77_HUMAN	NCBI
ARMC1	ARMC1_HUMAN	NCBI
HMGCLL1	HMGC2_HUMAN	NCBI
FHDC1	FHDC1_HUMAN	NCBI
NBEAL1	NBEL1_HUMAN	NCBI
RIMS4	RIMS4_HUMAN	NCBI
ZNF446	ZN446_HUMAN	NCBI
TRAM1L1	TR1L1_HUMAN	NCBI
GSG1L	GSG1L_HUMAN	NCBI
ATXN7L1	AT7L1_HUMAN	NCBI
NIPSNAP3B	NPS3B_HUMAN	NCBI
OR10J5	O10J5_HUMAN	NCBI
SNRNP48	SNR48_HUMAN	NCBI
KLHL23	KLH23_HUMAN	NCBI
PRSS55	PRS55_HUMAN	NCBI
LINC02914	CN177_HUMAN	NCBI
RNFT2	RNFT2_HUMAN	NCBI
DAPL1	DAPL1_HUMAN	NCBI
GPR141	GP141_HUMAN	NCBI
CRCT1	CRCT1_HUMAN	NCBI
HEPHL1	HPHL1_HUMAN	NCBI
C8orf34	CH034_HUMAN	NCBI
FBXO47	FBX47_HUMAN	NCBI
ATP8B3	AT8B3_HUMAN	NCBI
SUSD1	SUSD1_HUMAN	NCBI
SRL	SRCA_HUMAN	NCBI

CDH20	CAD20_HUMAN	NCBI
C11orf21	CK021_HUMAN	NCBI
TRIM67	TRI67_HUMAN	NCBI
TEX35	TEX35_HUMAN	NCBI
ZNF248	ZN248_HUMAN	NCBI
ODF3B	ODF3B_HUMAN	NCBI
KLHL31	KLH31_HUMAN	NCBI
UMAD1	UMAD1_HUMAN	NCBI
FYB2	FYB2_HUMAN	NCBI
ANKAR	ANKAR_HUMAN	NCBI
NUDT7	NUDT7_HUMAN	NCBI
MORN2	MORN2_HUMAN	NCBI
CCDC27	CCD27_HUMAN	NCBI
TPGS2	TPGS2_HUMAN	NCBI
SHISA6	SHSA6_HUMAN	NCBI
FSCB	FSCB_HUMAN	NCBI
C2CD4C	C2C4C_HUMAN	NCBI
ANKRD16	ANR16_HUMAN	NCBI
VSIG10	VSI10_HUMAN	NCBI
EEF1AKMT2	EFMT2_HUMAN	NCBI
PLD5	PLD5_HUMAN	NCBI
OR10J1	O10J1_HUMAN	NCBI
DEFB128	DB128_HUMAN	NCBI
TMEM229B	T229B_HUMAN	NCBI
HERPUD2	HERP2_HUMAN	NCBI
SPNS3	SPNS3_HUMAN	NCBI
DCDC2C	DCD2C_HUMAN	NCBI
ACBD7	ACBD7_HUMAN	NCBI
CRYBG2	CRBG2_HUMAN	NCBI
CYB561D1	C56D1_HUMAN	NCBI
CFAP77	CFA77_HUMAN	NCBI
CFAP299	CF299_HUMAN	NCBI
GASK1A	GAK1A_HUMAN	NCBI
GLB1L3	GLBL3_HUMAN	NCBI
ZNF470	ZN470_HUMAN	NCBI
CCDC13	CCD13_HUMAN	NCBI
FAM189A1	F1891_HUMAN	NCBI
PLEKHS1	PKHS1_HUMAN	NCBI
EML6	EMAL6_HUMAN	NCBI

SLC25A48	S2548_HUMAN	NCBI
ZNF585B	Z585B_HUMAN	NCBI
SAMD5	SAMD5_HUMAN	NCBI
FAM209B	F209B_HUMAN	NCBI
SAMD13	SAM13_HUMAN	NCBI
TMEM178A	T178A_HUMAN	NCBI
OR52M1	O52M1_HUMAN	NCBI
TRIM61	TRI61_HUMAN	NCBI
FAM185A	F185A_HUMAN	NCBI
C2orf42	CB042_HUMAN	NCBI
OR52K1	O52K1_HUMAN	NCBI
FAM180A	F180A_HUMAN	NCBI
C16orf96	CP096_HUMAN	NCBI
HSP90AA5P	HS905_HUMAN	NCBI
OR2A5	OR2A5_HUMAN	NCBI
C19orf18	CS018_HUMAN	NCBI
TP53TG3B	T53G3_HUMAN	NCBI
FLJ33534	YB035_HUMAN	NCBI
TMEM82	TMM82_HUMAN	NCBI
OR5T3	OR5T3_HUMAN	NCBI
AADACL4	ADCL4_HUMAN	NCBI
FAM218A	F218A_HUMAN	NCBI
TRAV40	TVA40_HUMAN	NCBI
LINC02912	TMM75_HUMAN	NCBI
LINC02085	YC023_HUMAN	NCBI
URGCP-MRPS24	S4R325_HUMAN	NCBI
CACTIN-AS1	CAAS1_HUMAN	NCBI
SLC6A14	S6A14_HUMAN	NCBI
MC3R	MC3R_HUMAN	NCBI
LEP	LEP_HUMAN	GeneCard
PRMT7	ANM7_HUMAN	GeneCard
SIM1	SIM1_HUMAN	GeneCard
BRCA2	BRCA2_HUMAN	GeneCard
ADCY3	ADCY3_HUMAN	GeneCard
CELA2A	CEL2A_HUMAN	GeneCard
BBS4	BBS4_HUMAN	GeneCard
BBS5	BBS5_HUMAN	GeneCard
MRAP2	MRAP2_HUMAN	GeneCard

OCA2	P_HUMAN	GeneCard
PRKAR1A	KAP0_HUMAN	GeneCard
TP53	P53_HUMAN	GeneCard
GHR	GHR_HUMAN	GeneCard
AKT1	AKT1_HUMAN	GeneCard
CYP19A1	CP19A_HUMAN	GeneCard
CDH23	CAD23_HUMAN	GeneCard
AKT2	AKT2_HUMAN	GeneCard
PTEN	PTEN_HUMAN	GeneCard
ALB	ALBU_HUMAN	GeneCard
WT1	WT1_HUMAN	GeneCard
IGFBP1	IBP1_HUMAN	GeneCard
PAX6	PAX6_HUMAN	GeneCard
TRH	TRH_HUMAN	GeneCard
FLT1	VGFR1_HUMAN	GeneCard
PRKACA	KAPCA_HUMAN	GeneCard
MECP2	MECP2_HUMAN	GeneCard
IAPP	IAPP_HUMAN	GeneCard
MYT1L	MYT1L_HUMAN	GeneCard
PHB1	PHB1_HUMAN	GeneCard
C3	CO3_HUMAN	GeneCard
MYO7A	MYO7A_HUMAN	GeneCard
ELP4	ELP4_HUMAN	GeneCard
SIRT1	SIR1_HUMAN	GeneCard
IL18	IL18_HUMAN	GeneCard
MET	MET_HUMAN	GeneCard
IL1B	IL1B_HUMAN	GeneCard
CEL	CEL_HUMAN	GeneCard
ACTB	ACTB_HUMAN	GeneCard
ACE	ACE_HUMAN	GeneCard
GAD2	DCE2_HUMAN	GeneCard
SLC2A1	GTR1_HUMAN	GeneCard
APOC3	APOC3_HUMAN	GeneCard
SIRT2	SIR2_HUMAN	GeneCard
IL4	IL4_HUMAN	GeneCard
GAPDH	G3P_HUMAN	GeneCard
CYP3A4	CP3A4_HUMAN	GeneCard
OFD1	OFD1_HUMAN	GeneCard
G6PC1	G6PC1_HUMAN	GeneCard

JUN	JUN_HUMAN	GeneCard
FABP3	FABPH_HUMAN	GeneCard
SIRT5	SIR5_HUMAN	GeneCard
SIRT7	SIR7_HUMAN	GeneCard
SIRT4	SIR4_HUMAN	GeneCard
BCHE	CHLE_HUMAN	GeneCard
PNLIP	LIPP_HUMAN	GeneCard
AKR1B1	ALDR_HUMAN	GeneCard
ADRA2A	ADA2A_HUMAN	GeneCard
LPIN1	LPIN1_HUMAN	GeneCard
MSTN	GDF8_HUMAN	GeneCard
PLTP	PLTP_HUMAN	GeneCard
FABP5	FABP5_HUMAN	GeneCard
DIO2	IOD2_HUMAN	GeneCard
APLN	APEL_HUMAN	GeneCard
CD4	CD4_HUMAN	GeneCard
TLR2	TLR2_HUMAN	GeneCard
GRP	GRP_HUMAN	GeneCard
DLEC1	DLEC1_HUMAN	GeneCard
MC1R	MSHR_HUMAN	GeneCard
HTR1A	5HT1A_HUMAN	GeneCard
SCARB2	SCRB2_HUMAN	GeneCard
CTSK	CATK_HUMAN	GeneCard
CFD	CFAD_HUMAN	GeneCard
AQP7	AQP7_HUMAN	GeneCard
IGF2R	MPRI_HUMAN	GeneCard
SUFU	SUFU_HUMAN	GeneCard
NOTCH3	NOTC3_HUMAN	GeneCard
HBA1	HBA_HUMAN	GeneCard
CCND1	CCND1_HUMAN	GeneCard
ERCC2	ERCC2_HUMAN	GeneCard
CNGB1	CNGB1_HUMAN	GeneCard
AHI1	AHI1_HUMAN	GeneCard
MSH2	MSH2_HUMAN	GeneCard
PDPN	PDPN_HUMAN	GeneCard
SLC27A3	S27A3_HUMAN	GeneCard
HBA2	HBA_HUMAN	GeneCard
RBP3	RET3_HUMAN	GeneCard
CYFIP1	CYFP1_HUMAN	GeneCard

PAPPA	PAPP1_HUMAN	GeneCard
BCL7B	BCL7B_HUMAN	GeneCard
SHH	SHH_HUMAN	GeneCard
FUZ	FUZZY_HUMAN	GeneCard
MYC	MYC_HUMAN	GeneCard
TIMP1	TIMP1_HUMAN	GeneCard
BRD4	BRD4_HUMAN	GeneCard
CLCN4	CLCN4_HUMAN	GeneCard
PRPF4	PRP4_HUMAN	GeneCard
KCNAB2	KCAB2_HUMAN	GeneCard
RP2	XRP2_HUMAN	GeneCard
ARVCF	ARVC_HUMAN	GeneCard
ADGRA3	AGRA3_HUMAN	GeneCard
PRCD	PRCD_HUMAN	GeneCard
ACE2	ACE2_HUMAN	GeneCard
FMO2	FMO2_HUMAN	GeneCard
GAD1	DCE1_HUMAN	GeneCard
BMP4	BMP4_HUMAN	GeneCard
CGA	GLHA_HUMAN	GeneCard
GLI1	GLI1_HUMAN	GeneCard
RTTN	RTTN_HUMAN	GeneCard
CXCL12	SDF1_HUMAN	GeneCard
DROSHA	RNC_HUMAN	GeneCard
PAX2	PAX2_HUMAN	GeneCard
CETN2	CETN2_HUMAN	GeneCard
DYNLT2B	DYT2B_HUMAN	GeneCard
STON1	STON1_HUMAN	GeneCard
TBX4	TBX4_HUMAN	GeneCard
DNMT1	DNMT1_HUMAN	GeneCard
CD274	PD1L1_HUMAN	GeneCard
TLR9	TLR9_HUMAN	GeneCard
MMP7	MMP7_HUMAN	GeneCard
CYCS	CYC_HUMAN	GeneCard
FGF4	FGF4_HUMAN	GeneCard
CYP17A1	CP17A_HUMAN	GeneCard
CFTR	CFTR_HUMAN	GeneCard
XIAP	XIAP_HUMAN	GeneCard
PRKAR2B	KAP3_HUMAN	GeneCard
TRIM37	TRI37_HUMAN	GeneCard

EPCAM	EPCAM_HUMAN	GeneCard
PCDH15	PCD15_HUMAN	GeneCard
AGTR1	AGTR1_HUMAN	GeneCard
DKK1	DKK1_HUMAN	GeneCard
FBXW7	FBXW7_HUMAN	GeneCard
TBX22	TBX22_HUMAN	GeneCard
HM13	HM13_HUMAN	GeneCard
NOG	NOGG_HUMAN	GeneCard
NTF3	NTF3_HUMAN	GeneCard
CELA3B	CEL3B_HUMAN	GeneCard
GNRHR	GNRHR_HUMAN	GeneCard
FGF3	FGF3_HUMAN	GeneCard
FHOD1	FHOD1_HUMAN	GeneCard
CDK6	CDK6_HUMAN	GeneCard
VPS35	VPS35_HUMAN	GeneCard
TLR3	TLR3_HUMAN	GeneCard
CST3	CYTC_HUMAN	GeneCard
SHOX2	SHOX2_HUMAN	GeneCard
JAG1	JAG1_HUMAN	GeneCard
TGFB2	TGFB2_HUMAN	GeneCard
ADA	ADA_HUMAN	GeneCard
VIM	VIME_HUMAN	GeneCard
TBX2	TBX2_HUMAN	GeneCard
CBX2	CBX2_HUMAN	GeneCard
RCN1	RCN1_HUMAN	GeneCard
DMRT2	DMRT2_HUMAN	GeneCard
DNAJC24	DJC24_HUMAN	GeneCard
OPRM1	OPRM_HUMAN	GeneCard
CYP2D6	CP2D6_HUMAN	GeneCard
CYP2C19	CP2CJ_HUMAN	GeneCard
PON2	PON2_HUMAN	GeneCard
GPX3	GPX3_HUMAN	GeneCard
PEG3	PEG3_HUMAN	GeneCard
HSPB1	HSPB1_HUMAN	GeneCard
MGP	MGP_HUMAN	GeneCard
ELANE	ELNE_HUMAN	GeneCard
GPX1	GPX1_HUMAN	GeneCard
CHI3L1	CH3L1_HUMAN	GeneCard
UTS2	UTS2_HUMAN	GeneCard

ASS1	ASSY_HUMAN	GeneCard
STAG2	STAG2_HUMAN	GeneCard
DPM1	DPM1_HUMAN	GeneCard
PCDH19	PCD19_HUMAN	GeneCard
CALB2	CALB2_HUMAN	GeneCard
KMT2B	KMT2B_HUMAN	GeneCard
PNPLA1	PLPL1_HUMAN	GeneCard
ASXL3	ASXL3_HUMAN	GeneCard
ESX1	ESX1_HUMAN	GeneCard
LDHA	LDHA_HUMAN	GeneCard
AQP1	AQP1_HUMAN	GeneCard
GP9	GPIX_HUMAN	GeneCard
POU5F1	PO5F1_HUMAN	GeneCard
CDKN2C	CDN2C_HUMAN	GeneCard
PKM	KPYM_HUMAN	GeneCard
MID1	TRI18_HUMAN	GeneCard
HPGDS	HPGDS_HUMAN	GeneCard
GP5	GPV_HUMAN	GeneCard
NUS1	NGBR_HUMAN	GeneCard
ANAPC2	ANC2_HUMAN	GeneCard
LRP5	LRP5_HUMAN	GeneCard
TNFRSF1A	TNR1A_HUMAN	GeneCard
MTCH2	MTCH2_HUMAN	GeneCard
GPR119	GP119_HUMAN	GeneCard
SELENOP	SEPP1_HUMAN	GeneCard
ARF4	ARF4_HUMAN	GeneCard
STRA8	STRA8_HUMAN	GeneCard
TM4SF20	T4S20_HUMAN	GeneCard
WNT3	WNT3_HUMAN	GeneCard
TBX21	TBX21_HUMAN	GeneCard
CHIT1	CHIT1_HUMAN	GeneCard
LTC4S	LTC4S_HUMAN	GeneCard
LGALS3BP	LG3BP_HUMAN	GeneCard
CSN2	CASB_HUMAN	GeneCard
KIAA0100	K0100_HUMAN	GeneCard
CTSB	CATB_HUMAN	GeneCard
AQP4	AQP4_HUMAN	GeneCard
DVL1	DVL1_HUMAN	GeneCard
BACE1	BACE1_HUMAN	GeneCard

EZR	EZRI_HUMAN	GeneCard
TFF1	TFF1_HUMAN	GeneCard
TSHB	TSHB_HUMAN	GeneCard
LIN28B	LN28B_HUMAN	GeneCard
EDC3	EDC3_HUMAN	GeneCard
MMUT	MUTA_HUMAN	GeneCard
MME	NEP_HUMAN	GeneCard
NEK8	NEK8_HUMAN	GeneCard
CETN3	CETN3_HUMAN	GeneCard
ASPM	ASPM_HUMAN	GeneCard
DGCR6	DGCR6_HUMAN	GeneCard
DGCR6L	DGC6L_HUMAN	GeneCard
BMP6	BMP6_HUMAN	GeneCard
CCL3	CCL3_HUMAN	GeneCard
HADHA	ECHA_HUMAN	GeneCard
PRKAR2A	KAP2_HUMAN	GeneCard
FOXP2	FOXP2_HUMAN	GeneCard
ADAM23	ADA23_HUMAN	GeneCard
RAD23A	RD23A_HUMAN	GeneCard
AP3B2	AP3B2_HUMAN	GeneCard
SSR2	SSRB_HUMAN	GeneCard
CABLES1	CABL1_HUMAN	GeneCard
POC1B	POC1B_HUMAN	GeneCard
PNPLA7	PLPL7_HUMAN	GeneCard
GSC2	GSC2_HUMAN	GeneCard
FLT4	VGFR3_HUMAN	GeneCard
SYK	KSYK_HUMAN	GeneCard
CYP1B1	CP1B1_HUMAN	GeneCard
AQP2	AQP2_HUMAN	GeneCard
BMP7	BMP7_HUMAN	GeneCard
NAT1	ARY1_HUMAN	GeneCard
APEX1	APEX1_HUMAN	GeneCard
NAT2	ARY2_HUMAN	GeneCard
IBSP	SIAL_HUMAN	GeneCard
PIM1	PIM1_HUMAN	GeneCard
VCP	TERA_HUMAN	GeneCard
YWHAG	1433G_HUMAN	GeneCard
MYH7	MYH7_HUMAN	GeneCard
HADHB	ECHB_HUMAN	GeneCard

HADH	HCDH_HUMAN	GeneCard
KCNA2	KCNA2_HUMAN	GeneCard
TMPRSS2	TMPS2_HUMAN	GeneCard
ADAMTS18	ATS18_HUMAN	GeneCard
KCNB2	KCNB2_HUMAN	GeneCard
GPX2	GPX2_HUMAN	GeneCard
SKIV2L	SKIV2_HUMAN	GeneCard
ALOXE3	LOXE3_HUMAN	GeneCard
FBL	FBRL_HUMAN	GeneCard
KCNAB1	KCAB1_HUMAN	GeneCard
KIFAP3	KIFA3_HUMAN	GeneCard
OTOF	OTOF_HUMAN	GeneCard
P2RX6	P2RX6_HUMAN	GeneCard
EXOSC3	EXOS3_HUMAN	GeneCard
EML1	EMAL1_HUMAN	GeneCard
AMY2B	AMY2B_HUMAN	GeneCard
RCBTB1	RCBT1_HUMAN	GeneCard
FIP1L1	FIP1_HUMAN	GeneCard
EXOSC9	EXOS9_HUMAN	GeneCard
ATAD3A	ATD3A_HUMAN	GeneCard
CD68	CD68_HUMAN	GeneCard
BOLL	BOLL_HUMAN	GeneCard
PROKR1	PKR1_HUMAN	GeneCard
OMP	OMP_HUMAN	GeneCard
DPY30	DPY30_HUMAN	GeneCard
LPCAT1	PCAT1_HUMAN	GeneCard
POF1B	POF1B_HUMAN	GeneCard
HARS1	HARS1_HUMAN	GeneCard
SMAP1	SMAP1_HUMAN	GeneCard
ADPRH	ADPRH_HUMAN	GeneCard
ABHD16A	ABHGA_HUMAN	GeneCard
GIMAP8	GIMA8_HUMAN	GeneCard
SMG9	SMG9_HUMAN	GeneCard
ACTRT2	ACTT2_HUMAN	GeneCard
EPPIN	EPPI_HUMAN	GeneCard
PRM2	PRM2_HUMAN	GeneCard
GET3	GET3_HUMAN	GeneCard
TSPY1	TSPY1_HUMAN	GeneCard
DAZ1	DAZ1_HUMAN	GeneCard

ZP4	ZP4_HUMAN	GeneCard
DPPA3	DPPA3_HUMAN	GeneCard
DBH	DOPO_HUMAN	GeneCard
KCNK9	KCNK9_HUMAN	GeneCard
IL4R	IL4RA_HUMAN	GeneCard
SLC12A1	S12A1_HUMAN	GeneCard
DCN	PGS2_HUMAN	GeneCard
CYSLTR2	CLTR2_HUMAN	GeneCard
HLA-A	HLAA_HUMAN	GeneCard
GZMB	GRAB_HUMAN	GeneCard
HTR3A	5HT3A_HUMAN	GeneCard
DUSP1	DUS1_HUMAN	GeneCard
DDR1	DDR1_HUMAN	GeneCard
MAP3K5	M3K5_HUMAN	GeneCard
TLR1	TLR1_HUMAN	GeneCard
WNT3A	WNT3A_HUMAN	GeneCard
HLA-C	HLAC_HUMAN	GeneCard
ACY1	ACY1_HUMAN	GeneCard
ALOX15	LOX15_HUMAN	GeneCard
CYP26B1	CP26B_HUMAN	GeneCard
PAX9	PAX9_HUMAN	GeneCard
HTR1F	5HT1F_HUMAN	GeneCard
FZD7	FZD7_HUMAN	GeneCard
GPNMB	GPNMB_HUMAN	GeneCard
MMP17	MMP17_HUMAN	GeneCard
CLCN1	CLCN1_HUMAN	GeneCard
BTC	BTC_HUMAN	GeneCard
B3GAT1	B3GA1_HUMAN	GeneCard
CYP26A1	CP26A_HUMAN	GeneCard
PRKD3	KPCD3_HUMAN	GeneCard
WNT2B	WNT2B_HUMAN	GeneCard
NOD1	NOD1_HUMAN	GeneCard
TLR6	TLR6_HUMAN	GeneCard
ZIC2	ZIC2_HUMAN	GeneCard
WNT7B	WNT7B_HUMAN	GeneCard
SLC4A2	B3A2_HUMAN	GeneCard
EREG	EREG_HUMAN	GeneCard
ABCC11	MRP8_HUMAN	GeneCard
INHA	INHA_HUMAN	GeneCard

CD82	CD82_HUMAN	GeneCard
KIRREL2	KIRR2_HUMAN	GeneCard
HAVCR1	HAVR1_HUMAN	GeneCard
STC2	STC2_HUMAN	GeneCard
CPA3	CBPA3_HUMAN	GeneCard
HTR1E	5HT1E_HUMAN	GeneCard
ADAM11	ADA11_HUMAN	GeneCard
STEAP4	STEA4_HUMAN	GeneCard
CST6	CYTM_HUMAN	GeneCard
TLR10	TLR10_HUMAN	GeneCard
ALX1	ALX1_HUMAN	GeneCard
BCAR3	BCAR3_HUMAN	GeneCard
TRERF1	TREF1_HUMAN	GeneCard
PRAME	PRAME_HUMAN	GeneCard
PSG1	PSG1_HUMAN	GeneCard
PNOC	PNOC_HUMAN	GeneCard
SPA17	SP17_HUMAN	GeneCard
CCNT2	CCNT2_HUMAN	GeneCard
BRMS1	BRMS1_HUMAN	GeneCard
ARID4B	ARI4B_HUMAN	GeneCard
EI24	EI24_HUMAN	GeneCard
ZIC5	ZIC5_HUMAN	GeneCard
YARS1	SYYC_HUMAN	GeneCard
ERVW-1	SYCY1_HUMAN	GeneCard
BRMS1L	BRM1L_HUMAN	GeneCard
KLK9	KLK9_HUMAN	GeneCard
SYT8	SYT8_HUMAN	GeneCard
PRRC2A	PRC2A_HUMAN	GeneCard
SCGB1D2	SG1D2_HUMAN	GeneCard
BEX2	BEX2_HUMAN	GeneCard
FASN	FAS_HUMAN	CTD
SREBF1	SRBP1_HUMAN	CTD
SOD1	SODC_HUMAN	CTD
FOS	FOS_HUMAN	CTD
PPARA	PPARA_HUMAN	CTD
TNF	TNFA_HUMAN	CTD
INS	INS_HUMAN	CTD
IL6	IL6_HUMAN	CTD
ICAM1	ICAM1_HUMAN	CTD

SOD2	SODM_HUMAN	CTD
CEBPA	CEBPA_HUMAN	CTD
PTGS2	PGH2_HUMAN	CTD
CCL2	CCL2_HUMAN	CTD
PCK1	PCKGC_HUMAN	CTD
DDIT3	DDIT3_HUMAN	CTD
LDLR	LDLR_HUMAN	CTD
NQO1	NQO1_HUMAN	CTD
AHR	AHR_HUMAN	CTD
PARP1	PARP1_HUMAN	CTD
SCD1	ACOD1_MOUSE	CTD
IRS1	IRS1_HUMAN	CTD
CRP	CRP_HUMAN	CTD
HSPA5	BIP_HUMAN	CTD
APOE	APOE_HUMAN	CTD
ACACB	ACACB_HUMAN	CTD
SERPINE1	PAI1_HUMAN	CTD
MMP9	MMP9_HUMAN	CTD
NR1I3	NR1I3_HUMAN	CTD
ACADM	ACADM_HUMAN	CTD
HSD11B1	DHI1_HUMAN	CTD
IGFBP2	IBP2_HUMAN	CTD
UCP2	UCP2_HUMAN	CTD
TFRC	TFR1_HUMAN	CTD
CASP1	CASP1_HUMAN	CTD
NR1I2	NR1I2_HUMAN	CTD
ACHE	ACES_HUMAN	CTD
IGF2	IGF2_HUMAN	CTD
FOXO3	FOXO3_HUMAN	CTD
NAMPT	NAMPT_HUMAN	CTD
VLDLR	VLDLR_HUMAN	CTD
ACLY	ACLY_HUMAN	CTD
SLC22A1	S22A1_HUMAN	CTD
OGG1	OGG1_HUMAN	CTD
PTPN1	PTN1_HUMAN	CTD
TF	TRFE_HUMAN	CTD
GLUL	GLNA_HUMAN	CTD
CS	CISY_HUMAN	CTD
TNFRSF1B	TNR1B_HUMAN	CTD

HTR2A	5HT2A_HUMAN	CTD
CIDEA	CIDEA_HUMAN	CTD
CD40	TNR5_HUMAN	CTD
HTR2C	5HT2C_HUMAN	CTD
ME1	MAOX_HUMAN	CTD
HMGB2	HMGB2_HUMAN	CTD
ITGAM	ITAM_HUMAN	CTD
CPE	CBPE_HUMAN	CTD
RAPGEF3	RPGF3_HUMAN	CTD
MRC1	MRC1_HUMAN	CTD
EP300	EP300_HUMAN	CTD
F2	THRB_HUMAN	CTD
SAT1	SAT1_HUMAN	CTD
LBP	LBP_HUMAN	CTD
SUCLG2	SUCB2_HUMAN	CTD
GNAS	GNAS3_HUMAN, GNAS2_HUMAN	CTD
HK2	HXK2_HUMAN	CTD
PYY	PYY_HUMAN	CTD
CYB5A	CYB5_HUMAN	CTD
SLC22A2	S22A2_HUMAN	CTD
GH1	SOMA_HUMAN	CTD
TRPV1	TRPV1_HUMAN	CTD
ECHS1	ECHM_HUMAN	CTD
CNR1	CNR1_HUMAN	CTD
ACSL1	ACSL1_HUMAN	CTD
PFKFB3	F263_HUMAN	CTD
SIRT3	SIR3_HUMAN	CTD
PEX11A	PX11A_HUMAN	CTD
DPYD	DPYD_HUMAN	CTD
ACP5	PPA5_HUMAN	CTD
DCXR	DCXR_HUMAN	CTD
IDO1	I23O1_HUMAN	CTD
CES1	EST1_HUMAN	CTD
GAS7	GAS7_HUMAN	CTD
CD163	C163A_HUMAN	CTD
NPY1R	NPY1R_HUMAN	CTD
COX7C	COX7C_HUMAN	CTD
ZFHX3	ZFHX3_HUMAN	CTD

AKR1C3	AK1C3_HUMAN	CTD
CPB2	CBPB2_HUMAN	CTD
ALDH6A1	MMSA_HUMAN	CTD
GNPDA2	GNPI2_HUMAN	CTD
FAAH	FAAH1_HUMAN	CTD
ALDH1L1	AL1L1_HUMAN	CTD
GFPT1	GFPT1_HUMAN	CTD
ADRB1	ADRB1_HUMAN	CTD
AKAP1	AKAP1_HUMAN	CTD
ATPAF1	ATPF1_HUMAN	CTD
UQCRC2	QCR2_HUMAN	CTD
GNB3	GBB3_HUMAN	CTD
EFNB1	EFNB1_HUMAN	CTD
PLIN1	PLIN1_HUMAN	CTD
ETFDH	ETFD_HUMAN	CTD
SLC22A3	S22A3_HUMAN	CTD
SUCLA2	SUCB1_HUMAN	CTD
AFF4	AFF4_HUMAN	CTD
LACTB	LACTB_HUMAN	CTD
RAB21	RAB21_HUMAN	CTD
SLC16A7	MOT2_HUMAN	CTD
CTF1	CTF1_HUMAN	CTD
TBC1D1	TBCD1_HUMAN	CTD
NHLH2	HEN2_HUMAN	CTD
HCRT	OREX_HUMAN	CTD
NEIL1	NEIL1_HUMAN	CTD
CA3	CAH3_HUMAN	CTD
APCDD1	APCD1_HUMAN	CTD
FTL	FRIL_HUMAN	CTD
ZBTB7B	ZBT7B_HUMAN	CTD
PMCH	MCH_HUMAN	CTD
INPP5E	INP5E_HUMAN	CTD
NPY5R	NPY5R_HUMAN	CTD
RAI1	RAI1_HUMAN	CTD
PCSK1	NEC1_HUMAN	CTD
PPM1L	PPM1L_HUMAN	CTD
ADH1B	ADH1B_HUMAN	CTD
GPR17	GPR17_HUMAN	CTD
ZFR2	ZFR2_HUMAN	CTD

APOA1	APOA1_HUMAN	CTD
ENTPD6	ENTP6_HUMAN	CTD
FCGR3B	FCG3B_HUMAN	CTD
ZNF169	ZN169_HUMAN	CTD
ADIPOQ	ADIPO_HUMAN	CTD
CETP	CETP_HUMAN	CTD
EIF2S3	IF2G_HUMAN	CTD
FFAR4	FFAR4_HUMAN	CTD
HERC2	HERC2_HUMAN	CTD
KSR2	KSR2_HUMAN	CTD
LAS1L	LAS1L_HUMAN	CTD
MAGEL2	MAGL2_HUMAN	CTD
MKRN3	MKRN3_HUMAN	CTD
MTTP	MTP_HUMAN	CTD
NDN	NECD_HUMAN	CTD
NPAP1	NPAP1_HUMAN	CTD
PHF6	PHF6_HUMAN	CTD
VPS13B	VP13B_HUMAN	CTD
ANGPTL4	ANGL4_HUMAN	CTD
UCP1	UCP1_HUMAN	CTD
PPARD	PPARD_HUMAN	CTD
FGF21	FGF21_HUMAN	CTD
IL7	IL7_HUMAN	CTD
GCG	GLUC_HUMAN	CTD
STS	STS_HUMAN	CTD
VGF	VGF_HUMAN	CTD
ZC3H10	ZC3HA_HUMAN	CTD
CCKAR	CCKAR_HUMAN	TTD
CCKBR	GASR_HUMAN	TTD
PCNA	PCNA_HUMAN	TTD
mGluR3	GRM3_HUMAN	TTD
mGluR2	GRM2_HUMAN	TTD
GOAT	MBOA4_HUMAN	TTD
SERT	SC6A4_HUMAN	TTD
NET	SC6A2_HUMAN	TTD
CHRM1	ACM1_HUMAN	TTD
CNTFR	CNTFR_HUMAN	TTD
PTAFR	PTAFR_HUMAN	TTD
METAP2	MAP2_HUMAN	TTD

GLP1R	GLP1R_HUMAN	TTD
DAT	SC6A3_HUMAN	TTD
OPRD1	OPRD_HUMAN	TTD
OPRK1	OPRK_HUMAN	TTD
DHCR24	DHC24_HUMAN	TTD
MCHR1	MCHR1_HUMAN	TTD
KCNA5	KCNA5_HUMAN	TTD
CDK1	CDK1_HUMAN	TTD
CDK5	CDK5_HUMAN	TTD
CDK2	CDK2_HUMAN	TTD
ADRA2C	ADA2C_HUMAN	TTD
PCAT	PCAT1_HUMAN, PCAT2_HUMAN	TTD
NPY2R	NPY2R_HUMAN	TTD
TPP2	TPP2_HUMAN	TTD
DGAT1	DGAT1_HUMAN	TTD
GHSR	GHSR_HUMAN	TTD
NPY4R	NPY4R_HUMAN	TTD
BRS3	BRS3_HUMAN	TTD
CPT1B	CPT1B_HUMAN	TTD
HTR6	5HT6R_HUMAN	TTD
MAO-B	AOFB_HUMAN	TTD
AMPK	AAPK1_HUMAN, AAKB1_HUMAN, AAKG1_HUMAN	TTD
H3R	HRH3_HUMAN	TTD
LIPE	LIPS_HUMAN	TTD
PH	PAHO_HUMAN	TTD
SLC22A8	S22A8_HUMAN	TTD
H1R	HRH1_HUMAN	TTD
FGFR4 mRNA	FGFR4_HUMAN	TTD
ADRA1B	ADA1B_HUMAN	TTD

Supplementary Table S2.

Name	Degree	Genes target
**Kaempferol**	114	ABCB1, ABCC1, ABCG2, ACHE, ADORA1, ADORA2A, AHR, AKR1A1, AKR1B1, AKR1B10, AKR1C1, AKR1C2, AKR1C3, AKR1C4, AKT1, ALK, ALOX12, ALOX15, ALOX5, AMY1A, APP, ARG1, AT1G06000, AURKB, AVPR2, AXL, BACE1, C4H, CA1, CA12, CA13, CA14, CA2, CA3, CA4, CA5A, CA6, CA7, CA9, CAMK2B, CCNB1, CCNB2, CCNB3, CD38, CDK1, CDK1, CDK2, CDK5, CDK5R1, CDK6, CFTR, CSNK2A1, CXCR1, CYP19A1, CYP1B1, DAPK1, DRD4, EGFR, ESR1, ESR2, ESRRA, F2, FLS1, FLS3, FLS4, FLS5, FLS6, FLT3, GLO1, GPR35, GRK6, GSK3B, HSD17B1, HSD17B2, IGF1R, KDR, MAOA, MAPT, MET, MMP12, MMP13, MMP2, MMP3, MMP9, MPG, MPO, NEK2, NEK6, NOX4, NUAK1, PARP1, PFKFB3, PIK3R1, PIM1, PKN1, PLA2G1B, PLK1, PTGS2, PTK2, PTPRS, PYGL, SLC22A12, SRC, SYK, TERT, TNKS, TNKS2, TOP1, TT7, TTR, TYR, UGT78D1, UGT78D2, XDH
**Cheilanthifoline**	113	ABCB1, ABL1, ADORA1, ADORA2A, ADORA2B, ADRA1A, ADRA1B, ADRA1D, ADRA2A, ADRA2C, ADRB1, ADRB2, ADRB3, AKR1B1, ALOX5, ANPEP, APH1A, APH1B, APP, AR, BCHE, BRAF, CA12, CCNA1, CCNA2, CCNB1, CCNB2, CCNB3, CDK1, CDK1, CDK2, CDK2, CDK4, CDK5, CDK5R1, CHEK1, CHEK2, CHRM4, CHRNA2, CHRNA3, CHRNA4, CHRNB2, CHRNB4, CLK1, COMT, CYP19A1, DCTPP1, DHCR7, DNM1, DRD1, DRD2, DRD3, DRD4, DRD5, DYRK1A, DYRK1B, EGFR, ENPP2, ERN1, ESR1, ESR2, ESRRA, ESRRB, F3, GCGR, HCRTR1, HSD17B1, HSD17B2, HSD17B3, HSP90AA1, HTR1A, HTR2A, HTR2B, HTR7, ILK, KCNN1, KCNN2, KIF11, MAOA, MAOB, MAP2K1, MAPKAPK2, MMP2, MTNR1A, MTNR1B, NCSTN, NEK1, PARP2, PIK3CG, PIM1, PSEN1, PSEN2, PSENEN, PTGS1, QDPR, RAF1, RBBP9, SERPINE1, SHBG, SIGMAR1, SLC18A2, SLC47A1, SLC6A3, SRC, STS, SYK, TBK1, TBXA2R, TRPM8, TUBB1, TUBB3, TYMS, WEE1
**Quercetin**	113	ABCB1, ABCC1, ABCG2, ACHE, ADORA1, ADORA2A, AHR, AKR1A1, AKR1B1, AKR1B10,

		AKR1C1, AKR1C2, AKR1C3, AKR1C4, AKT1, ALK, ALOX12, ALOX15, ALOX5, APEX1, APP, ARG1, AURKB, AVPR2, AXL, BACE1, CA1, CA12, CA13, CA14, CA2, CA3, CA4, CA5A, CA6, CA7, CA9, CAMK2B, CCNB1 , CCNB2, CCNB3 , CD38, CDK1, CDK1 , CDK2, CDK5, CDK5R1 , CDK6, CSNK2A1, CXCR1, CYP19A1, CYP1B1, DAPK1, DRD4, EGFR, ESR2, ESRRA, F2, FLT3, GLO1, GPR35, GSK3B, HSD17B1, HSD17B2, IGF1R, INSR, KDM4E, KDR, MAOA, MAPT, MET, MMP12, MMP13, MMP2, MMP3, MMP9, MPG, MPO, MYLK, NEK2, NEK6, NOX4, NUAK1, OsI_09072, OsI_15081, OsI_15082, OsI_21986, OsI_26177, OsI_27880, OsI_33044, OsI_33047, PARP1, PIK3CG, PIK3R1, PIM1, PKN1, PLA2G1B, PLK1, PTK2, PTPRS, PYGL, SLC22A12, SRC, SYK, TERT, TNKS, TNKS2, TOP1, TOP2A, TTR, TYR, xd1, XDH
**(R)-(6-methoxy-4-quinolyl)-[(2R,4R,5S)-5-vinylquinuclidin-2-yl]methanol**	107	ABCB1, ABL1, ADORA2A, ADORA3, ADRA1A, ADRB1, ADRB2, ADRB3, ALK, AR, ATAD2, BAZ2A, BAZ2B, BCHE, BIRC2, BLK, BRD2, BRD4, CA1, CA2, CA9, CALCRL, CAMK2G, CASR, CDK6, CHRM1, CHRM2, CHRM3, CHRNA2, CHRNA3, CHRNA3, CHRNA4, CHRNA5, CHRNA6, CHRNB2, CHRNB2, CHRNB3, CHRNB4, DPP7, DPP8, DPP9, DRD1, DRD2, DRD3, DRD4, ERBB2, FGFR3, FLT1, GSK3A, H1F0, HSP90AA1, HTR1A, HTR1B, HTR1D, HTR2A, HTR6, INSR, JAK1, KCNA5, KCNH2, KCNK2, KIT, LAP3, LCK, LYN, MAP2K1, MAP3K12, MAP3K5, MAPK11, MST1R, MTAP, MTOR, NEK1, NOS2, OGFRL1, OPRK1, OPRM1, PDE1C, PDE7A, PDE9A, PEPD, PIK3CA, PIM2, PIM3, PLK2, PNMT, PRKCA, PRKCB, PRKCE, PRKCG, PRKCQ, PRKCZ, ROCK1, ROCK2, RPS6KA1, SCN5A, SIGMAR1, SLC6A4, SLC6A9, SRC, SYK, TEK, TERT, TRPV3, WNK2, XIAP, ZAP70
**Luteolin**	105	ABCB1, ABCC1, ABCG2, ACHE, ADORA1, ADORA2A, AHR, AKR1A1, AKR1B1, AKR1B10, AKR1C1, AKR1C2, AKR1C3, AKR1C4, AKT1, ALK, ALOX12, ALOX15, ALOX5, AMY1A, APP, AR, ARG1, AURKB, AVPR2, AXL, BACE1, CA1, CA12, CA13, CA14, CA2, CA3, CA4, CA5A, CA6, CA7, CA9, CAMK2B, CCNB1, CCNB2, CCNB3, CD38, CDK1, CDK1, CDK2, CDK5, CDK5R1, CDK6, CFTR, CSNK2A1, CXCR1,

		CYP19A1, CYP1B1, DAPK1, DRD4, EGFR, ESR1, ESR2, ESRRA, F2, FLT3, GLO1, GPR35, GRK6, GSK3B, HSD17B1, HSD17B2, HY5, IGF1R, KDM4E, KDR, MAOA, MET, MMP12, MMP13, MMP2, MMP3, MMP9, MPO, NEK2, NEK6, NOX4, NUAK1, PARP1, PFKFB3, PIK3R1, PIM1, PKN1, PLA2G1B, PLG, PLK1, PTGS2, PTK2, PTPRS, PYGL, SRC, SYK, TERT, TNKS, TNKS2, TOP1, TTR, TYR, XDH
**Phyllanthin**	105	ABL1, ADORA1, ADORA2A, AKT1, ALOX12, ALOX15, ALOX5, AR, AURKA, AURKB, AVPR1B, CA1, CA2, CA4, CA6, CCNC , CCNE1, CCNE2 , CDK2 , CDK8, CHEK1, CNR1, CNR2, CRHR1, CYP19A1, CYP24A1, ERBB2, FKBP5, FLT1, FNTA, FNTB, GABRA1, GABRA2, GABRA3, GABRA5, GABRB3, GABRG2, GABRG2, GCK, GPBAR1, GPR119, GRM1, GRM4, GRM5, GSK3B, HCRTR1, HIPK1, HSP90B1, HTR1A, IGF1R, JAK1, JAK2, JAK3, KDR, LYN, LYPLA1, LYPLA2, MAP2K1, MAP3K12, MAPK10, MAPK8, MDM2, MTOR, NR3C1, NTRK1, OXTR, PDE10A, PDE11A, PDE2A, PDE3A, PDE3B, PDE4A, PDE4B, PDE4D, PDE9A, PDPK1, PIK3CA, PIK3CB, PIK3CD, PIK3CG, PIM1, POLA1, PPARG, PRKDC, PTAFR, PTGER3, PTGFR, ROS1, RPS6KB1, SHBG, SLC6A3, SLC6A5, SLC6A9, SMO, STS, SYK, TGFBR1, TLR9, TNFRSF1A, TNK2, TRAP1, TTR, TYK2, UTS2R, YES1
**Peraksine**	74	ABCB1, ABCG2, ACHE, ADORA3, ADRA1A, ADRA1B, ADRA1D, ADRA2A, ADRA2B, ADRA2C, ADRB3, AKT1, BAZ2A, BAZ2B, BCHE, CA1, CA12, CA13, CA7, CA9, CHEK1, CHRM4, CHRNA2, CHRNA3, CHRNA3, CHRNA4, CHRNA5, CHRNA6, CHRNB2, CHRNB2, CHRNB3, CHRNB4, CTSC, CYP2D6, DPP7, DRD1, DRD2, DRD3, FDFT1, GSK3A, GSK3B, HTR1A, HTR1F, HTR2A, HTR2B, HTR6, HTR7, ILK, KCNH2, KISS1R, LRRK2, MCHR1, NOS1, NOS2, NOS3, OPRD1, OPRK1, OPRL1, OPRM1, PARP1, ROCK1, ROCK2, RPS6KA5, SIGMAR1, SLC6A2, SLC6A3, SLC6A4, SLC6A9, SSTR3, TLR4, TNNC1, TNNI3, TNNT2, UTS2R
**Ellagic acid**	70	AKR1B1, AKT1, ALOX5, AURKA, AURKB, BACE1, BRAF, CA1, CA12, CA13, CA14, CA2, CA4, CA5A, CA5B, CA6, CA7, CA9, CBR1,

		CCNA1, CCNA2, CCND1, CDK2, CDK4, CDK5, CDK5R1, CES2, CSNK2A1, DAO, EGFR, EPHB4, ERBB2, ESR1, ESR2, F12, FGR, FLT4, GPR35, GSK3B, GSR, HSD17B3, HSPA1A, IGF1R, IL4, INSR, KDR, LYN, MAOA, MAP3K8, MET, NME1-NME2, NME2, NOS3, NUAK1, PDGFRB, PLK1, PLK4, PTGS2, PTK2, PTPN1, SNCA, SQLE, SRC, SYK, TEK, TNNC1, TNNI3, TNNT2, TYR, XDH
**Leucodelphinidin**	60	ABCB1, all2390, all2598, all4752, all4874, all5026, all5295, all5305, ALOX12, ALOX15, alr2751, alr2903, alr4268, APP, BACE1, BCL2, CA1, CA12, CA13, CA2, CA3, CA4, CA5A, CA5B, CA6, CA7, CA9, CYP1B1, DNMT1, DYRK1A, ESR1, FGFR1, FUT4, FUT7, GABRA1, GABRB2, GABRG2, HIF1A, KCNH2, KDR, KIT, KLK1, KLK2, MAPK14, MAPT, MET, MMP12, MMP13, MMP14, MMP2, MMP9, PGD, PGF, PTGS1, SQLE, SRC, ST3GAL3, STAT1, TERT, VEGFA
**α-amyrin**	59	ACHE, ACP1, ADORA3, AKR1B10, ALOX5, AR, BCHE, CD81, CDC25A, CDC25B, CES2, CHRM2, CNR1, CYP17A1, CYP19A1, CYP2C19, CYP51A1, ESR1, ESR2, FAAH, FABP1, FABP3, FABP4, FABP5, FNTA, FNTB, HMGCR, HSD11B1, HSD11B2, MAPK3, NOS2, NPC1L1, NR1H3, NR1I3, PDE4D, PLA2G1B, POLB, PPARA, PPARD, PPARG, PREP, PRKCH, PTGES, PTPN1, PTPN11, PTPN2, PTPN6, PTPRF, RORA, RORC, SCD, SERPINA6, SHBG, SLC6A2, SLC6A4, SQLE, SREBF2, TERT, UGT2B7
**7-Dehydrosigmasterol**	56	ACHE, ACP1, ADORA3, AKR1B10, AR, ATP12A, BACE1, BCHE, CDC25A, CDC25B, CES2, CHRM2, CNR1, CYP17A1, CYP19A1, CYP2C19, CYP51A1, DHCR7, ESR1, ESR2, FABP1, FABP3, FABP4, FABP5, G6PD, GLRA1, HMGCR, HSD11B1, HSD11B2, MAPK3, NOS2, NPC1L1, NR1H2, NR1H3, NR1I3, PLA2G1B, POLB, PPARA, PPARD, PPARG, PREP, PTGES, PTPN1, PTPN11, PTPN6, PTPRF, RORA, RORC, SERPINA6, SHBG, SLC6A2, SLC6A4, SQLE, SREBF2, TOP2A, VDR
**(-)-Epigallocatechin-3-gallate**	47	ABCB1, ABCC1, ABCG2, APP, BACE1, BCL2, CA1, CA12, CA13, CA2, CA3, CA4, CA5A, CA5B, CA6, CA7, CA9, CYP1B1, DNM1, DNMT1, DYRK1A, FUT4, FUT7, GABRA1, GABRB2, GABRG2, HIF1A, KCNH2, KLK1,

		KLK2, MAPK14, MAPT, MET, MMP12, MMP13, MMP14, MMP2, MMP9, PGD, PGF, PTGS1, SQLE, ST3GAL3, STAT1, TAS2R31, TERT, VEGFA
**Beta-sitosterol**	44	ACHE, AR, BCHE, CDC25A, CDC25B, CES2, CHRM2, CYP17A1, CYP19A1, CYP2C19, CYP51A1, DHCR7, DRD2, ESR1, ESR2, FDFT1, G6PD, GLRA1, HMGCR, HSD11B1, HSD11B2, NOS2, NPC1L1, NR1H2, NR1H3, NR1I3, NR3C1, POLB, PPARD, PTGER1, PTGER2, PTPN1, PTPN6, RORA, RORC, SERPINA6, SHBG, SHH, SLC6A2, SLC6A4, SQLE, SREBF2, UGT2B7, VDR
**Ellipticine**	41	ABCC1, ALDH2, CCNB1, CCNB2, CCNB3, CCR1, CCR5, CCR8, CDK1, CTSK, CTSL, CTSS, Cyp1a1, CYP24A1, CYP26A1, CYP26B1, ENSRNOG00000046657, ERBB2, F2R, FGFR3, FNTA, FNTB, GSK3B, GSTP1, HRH3, HTR2C, IMPDH2, JAK1, JAK3, KIF11, KIT, Kit, PDGFRA, PLA2G7, PTK6, QPCT, STAT3, TAAR1, TOP2A, Top2a, Tp53
**(2S,3R,3aS,4R,4'S,5'R,6S,7aR)-3,4,4'-trihydroxy-3,5'-bis(hydroxymethyl)spiro[3a,4,5,6,7,7a-hexahydrobenzofuran-2,2'-tetrahydropyran]-6-carboxylic acid**	33	ADRA1A, ADRA1D, ADRA2A, ADRA2B, ADRA2C, APH1A, APH1B, CDK1, CYP2D6, DRD1, DRD2, DRD3, FGF2, GLRA1, GLRA2, HPSE, HTR1B, HTR2A, HTR2B, HTR2C, HTR6, NCSTN, OPRK1, PPM1A, PSEN1, PSEN2, PSENEN, PTAFR, RORC, SELL, STAT3, VDR, VEGFA
**Digallate**	24	CA1, CA12, CA14, CA2, CA3, CA5A, CA6, CA7, CA9, CADAFLAP00004221, CADAFLAP00007429, CADAFLAP00012628, CADAFLAP00013265, CSNK2A1, CSNK2A2, faeB-1, faeB-2, FUT7, MAOA, MAOB, POLA1, POLB, SERPINE1, TUBB1
**(+)-Catechin**	9	APOB, BACE1, CSF2, DNMT1, HMOX1, PON1, PTGS2, SLC22A11, SLC47A1
**Mucic acid 1,4-lactone 5-0-gallate**	9	ABCB1, ACHE, BACE1, F10, PTPN1, PTPN2, SBH1, SERPINE1, SQLE
**Sennidin C**	1	FTO

Supplementary Table S3.

Name	Degree	BetweennessCentrality	ClosenessCentrality
AKT1	42	0.16	0.60
PPARG	33	0.11	0.57
PTGS2	32	0.07	0.56
ESR1	29	0.10	0.53
MMP9	25	0.04	0.50
PPARA	25	0.05	0.53
CCND1	24	0.06	0.49
TLR4	22	0.05	0.52
SLC6A4	22	0.08	0.51
CNR1	20	0.07	0.52
SLC6A3	19	0.05	0.50
IL4	18	0.01	0.49
CYP2D6	18	0.04	0.49
HMOX1	17	0.01	0.47
ADRB2	16	0.04	0.51
CYP19A1	15	0.02	0.48
AHR	15	0.02	0.49
XIAP	14	0.01	0.45
CDK2	14	0.00	0.46
SERPINE1	14	0.00	0.47
TNFRSF1A	14	0.02	0.46
SLC6A2	14	0.01	0.42
MAOB	14	0.01	0.42
CDK1	13	0.01	0.43
CYP2C19	13	0.02	0.47
ACHE	13	0.03	0.49
CDK6	12	0.00	0.43
F2	12	0.02	0.47
HTR2C	12	0.01	0.43
HTR1A	12	0.01	0.42
CDK5	12	0.02	0.44
NOX4	11	0.00	0.44
CHRM1	11	0.02	0.39
HTR2A	10	0.01	0.44
UGT2B7	10	0.01	0.39
CYP1B1	10	0.01	0.45

FAAH	10	0.02	0.45
OPRM1	9	0.01	0.42
PARP1	9	0.01	0.42
DNMT1	9	0.00	0.44
ARG1	9	0.00	0.43
LRRK2	9	0.01	0.46
RPS6KB1	9	0.00	0.45
AKR1C3	9	0.01	0.43
PTPN1	9	0.00	0.44
AKR1B1	9	0.00	0.43
CFTR	9	0.02	0.42
TLR9	8	0.00	0.42
SHH	8	0.00	0.42
BACE1	8	0.01	0.45
SYK	7	0.01	0.44
DRD1	7	0.01	0.40
MET	7	0.00	0.43
BCHE	7	0.00	0.40
CYP17A1	7	0.00	0.39
CTSK	7	0.00	0.42
ADRB1	7	0.01	0.45
ADRA1B	7	0.00	0.40
CHRNA3	7	0.01	0.40
HSD11B1	6	0.00	0.42
FLT1	6	0.00	0.41
ATP12A	6	0.00	0.42
ALOX15	6	0.00	0.44
FABP5	6	0.01	0.40
NR1I3	6	0.02	0.37
ADRA2C	6	0.01	0.38
ADRA2A	6	0.01	0.38
HRH3	6	0.02	0.36
FABP3	5	0.00	0.39
CYP26B1	5	0.00	0.35
MAP3K5	5	0.00	0.40
PPARD	5	0.00	0.42
BRD4	5	0.00	0.41
XDH	5	0.00	0.39
OPRD1	5	0.00	0.38

ADRB3	5	0.00	0.40
CTSS	4	0.00	0.37
MTNR1B	4	0.00	0.40
CYP26A1	4	0.00	0.34
STS	4	0.00	0.37
OPRK1	3	0.00	0.36
TNKS	3	0.00	0.34
APEX1	3	0.00	0.37
FTO	3	0.00	0.37
PDE4D	3	0.00	0.40
ADORA3	3	0.00	0.43
PTAFR	2	0.00	0.35
HTR1F	2	0.00	0.35
FLT4	2	0.00	0.34
GPR119	2	0.00	0.36
MTNR1A	2	0.00	0.34
CHRM3	2	0.00	0.31
HTR6	2	0.00	0.37
PFKFB3	2	0.00	0.38
PRKCH	2	0.00	0.28
KCNN2	1	0.00	1.00
KCNA5	1	0.00	1.00
MCHR1	1	0.00	0.26
PIM1	1	0.00	0.33
DYRK1B	1	0.00	0.33
CA3	1	0.00	0.27

Supplementary Table S4.

Name	Degree	Genes target
Luteolin	28	AKT1, ESR1, PTGS2, MMP9, CYP19A1, ACHE, CDK2, CDK1, AHR, BACE1, CDK5, CDK6, CYP1B1, F2, AKR1B1, NOX4, SYK, MET, PARP1, ARG1, AKR1C3, CFTR, ALOX15, XDH, CA3, TNKS, PIM1, PFKFB3
Kaempferol	28	AKT1, ESR1, PTGS2, MMP9, CYP19A1, ACHE, CDK2, CDK1, AHR, BACE1, CDK5, CDK6, CYP1B1, F2, AKR1B1, NOX4, SYK, MET, PARP1, ARG1, AKR1C3, CFTR, ALOX15, XDH, CA3, TNKS, PIM1, PFKFB3
Cheilanthifoline	27	ESR1, CYP19A1, SLC6A3, CDK2, CDK1, ADRB2, CDK5, SERPINE1, HTR1A, MAOB, AKR1B1, HTR2A, SYK, BCHE, DRD1, ADRA1B, ADRB1, ADRA2A, ADRA2C, ADRB3, PIM1, STS, MTNR1B, CHRNA3, MTNR1A, DYRK1B, KCNN2
Quercetin	25	AKT1, MMP9, CYP19A1, ACHE, CDK2, CDK1, AHR, BACE1, CDK5, CDK6, CYP1B1, F2, AKR1B1, NOX4, SYK, MET, PARP1, ARG1, AKR1C3, ALOX15, XDH, CA3, TNKS, PIM1, APEX1
Peraksine	25	AKT1, SLC6A4, TLR4, SLC6A3, ACHE, CYP2D6, SLC6A2, HTR1A, HTR2A, BCHE, PARP1, DRD1, OPRM1, LRRK2, ADRA1B, ADRA2A, ADRA2C, ADRB3, ADORA3, OPRK1, OPRD1, HTR6, CHRNA3, HTR1F, MCHR1
α-amyrin	22	ESR1, PPARG, PPARA, SLC6A4, CNR1, CYP19A1, ACHE, SLC6A2, CYP2C19, PTPN1, BCHE, UGT2B7, FAAH, CYP17A1, HSD11B1, NR1I3, FABP5, PPARD, FABP3, ADORA3, PDE4D, PRKCH
(R)-(6-methoxy-4-quinolyl)-[(2R,4R,5S)-5-vinylquinuclidin-2-yl]methanol	22	SLC6A4, ADRB2, CDK6, HTR1A, XIAP, HTR2A, SYK, BCHE, CHRM1, DRD1, OPRM1, ADRB1, FLT1, ADRB3, ADORA3, BRD4, MAP3K5, OPRK1, HTR6, CHRNA3, CHRM3, KCNA5
7-Dehydrosigmasterol	20	ESR1, PPARG, PPARA, SLC6A4, CNR1, CYP19A1, ACHE, SLC6A2, BACE1, CYP2C19, PTPN1, BCHE, CYP17A1, HSD11B1, NR1I3, FABP5, PPARD, ATP12A, FABP3, ADORA3
Phyllanthin	18	AKT1, PPARG, CNR1, CYP19A1, SLC6A3, CDK2, HTR1A, TNFRSF1A, SYK, ALOX15,

		TLR9, FLT1, PIM1, STS, PDE4D, PTAFR, GPR119, SLC6A5,
Beta-sitosterol	14	ESR1, SLC6A4, CYP19A1, ACHE, SLC6A2, CYP2C19, PTPN1, BCHE, UGT2B7, CYP17A1, HSD11B1, NR1I3, SHH, PPARD
Ellagic acid	14	AKT1, ESR1, PTGS2, CDK2, IL4, BACE1, CDK5, AKR1B1, SYK, PTPN1, MET, XDH, FLT4, CCND1
(2S,3R,3aS,4R,4'S,5'R,6S,7aR)-3,4,4'-trihydroxy-3,5'-bis(hydroxymethyl)spiro[3a,4,5,6,7,7a-hexahydrobenzofuran-2,2'-tetrahydropyran]-6-carboxylic acid	10	CYP2D6, CDK1, HTR2A, HTR2C, DRD1, ADRA2A, ADRA2C, OPRK1, HTR6, PTAFR
Leucodelphinidin	8	ESR1, MMP9, BACE1, CYP1B1, MET, DNMT1, ALOX15, CA3
Ellipticine	7	CDK1, HTR2C, CTSK, HRH3, CYP26B1, CYP26A1, CTSS
(-)-Epigallocatechin-3-gallate	6	MMP9, BACE1, CYP1B1, MET, DNMT1, CA3
(+)-Catechin	5	PTGS2, HMOX1, BACE1, DNMT1, SLC22A11
Mucic acid 1,4-lactone 5-0-gallate	4	ACHE, BACE1, SERPINE1, PTPN1
Digallate	3	SERPINE1, MAOB, CA3
7-Dehydrosigmasterol	1	FTO

Supplementary Table S5.

KEGG pathway	Degree	Genes target
Neuroactive ligand-receptor interaction	26	MTNR1B, ADRA2A, HTR2A, CHRM1, ADRB3, DRD1, ADRB2, MCHR1, PTAFR, ADRB1, HTR2C, HRH3, ADORA3, ADRA1B, CHRNA3, HTR6, HTR1A, OPRK1, ADRA2C, CHRM3, OPRD1, F2, OPRM1, MTNR1A, HTR1F, CNR1
Pathways in cancer	18	PIM1, CCND1, PPARD, AKT1, FLT4, PTGS2, PPARG, MMP9, MET, F2, CDK6, CDK2, XIAP, ESR1, HMOX1, SHH, IL4, RPS6KB1
Calcium signaling pathway	14	HTR2C, HTR2A, CHRM1, ADRB3, FLT4, ADRA1B, HTR6, MET, CHRM3, DRD1, FLT1, ADRB2, PTAFR, ADRB1
Chemical carcinogenesis - receptor activation	14	AHR, CCND1, NR1I3, CYP1B1, ADRB3, AKT1, CHRNA3, UGT2B7, PPARA, ADRB2, XIAP, ESR1, RPS6KB1, ADRB1
PI3K-Akt signaling pathway	12	TLR4, SYK, CCND1, CHRM1, MET, CDK6, FLT1, FLT4, AKT1, CDK2, IL4, RPS6KB1
cAMP signaling pathway	12	GPR119, CHRM1, DRD1, PPARA, AKT1, CFTR, ADRB2, HTR6, HTR1A, HTR1F, PDE4D, ADRB1
Serotonergic synapse	12	SLC6A4, HTR2C, HTR2A, CYP2D6, CYP2C19, ALOX15, HTR6, HTR1A, HTR1F, PTGS2, MAOB, KCNN2
Chemical carcinogenesis - reactive oxygen species	9	AHR, MET, AKR1C3, CYP1B1, MAP3K5, PTPN1, NOX4, AKT1, HMOX1
cGMP-PKG signaling pathway	9	ADRA2A, ADRA2C, OPRD1, ADRB3, ADORA3, AKT1, ADRA1B, ADRB2, ADRB1
Proteoglycans in cancer	8	TLR4, CCND1, MMP9, MET, AKT1, ESR1, SHH, RPS6KB1
Alcoholic liver disease	7	TLR4, TNFRSF1A, CCND1, PPARA, MAP3K5, NOX4, AKT1
Apoptosis	7	TNFRSF1A, MAP3K5, AKT1, XIAP, CTSS, CTSK, PARP1
HIF-1 signaling pathway	7	TLR4, PFKFB3, FLT1, AKT1, SERPINE1, HMOX1, RPS6KB1
Steroid hormone biosynthesis	7	UGT2B7, CYP17A1, AKR1C3, CYP1B1, CYP19A1, STS, HSD11B1
AMPK signaling pathway	6	PPARG, CCND1, PFKFB3, AKT1, CFTR, RPS6KB1
TNF signaling pathway	6	TNFRSF1A, MMP9, MAP3K5, AKT1, XIAP, PTGS2
NF-kappa B signaling pathway	6	TLR4, TNFRSF1A, SYK, XIAP, PTGS2, PARP1

Endocrine resistance	6	CCND1, MMP9, CYP2D6, AKT1, ESR1, RPS6KB1
Small cell lung cancer	6	CCND1, CDK6, AKT1, CDK2, XIAP, PTGS2
PPAR signaling pathway	5	PPARG, FABP3, FABP5, PPARD, PPARA
p53 signaling pathway	5	CCND1, CDK6, CDK2, CDK1, SERPINE1
Chemical carcinogenesis - DNA adducts	5	UGT2B7, CYP1B1, CYP2C19, PTGS2, HSD11B1
Acute myeloid leukemia	5	PIM1, CCND1, PPARD, AKT1, RPS6KB1
Regulation of lipolysis in adipocytes	5	ADRB3, AKT1, ADRB2, PTGS2, ADRB1
Ovarian steroidogenesis	5	CYP17A1, AKR1C3, CYP1B1, CYP19A1, PTGS2
